# Inferring intra-motif dependencies of DNA binding sites from ChIP-seq data

**DOI:** 10.1186/s12859-015-0797-4

**Published:** 2015-11-09

**Authors:** Ralf Eggeling, Teemu Roos, Petri Myllymäki, Ivo Grosse

**Affiliations:** 1 Institute of Computer Science, Martin Luther University Halle-Wittenberg, Halle, Germany; 2 Department of Computer Science, Helsinki Institute for Information Technology HIIT, University of Helsinki, Helsinki, Finland; 3 German Center for Integrative Biodiversity Research (iDiv) Halle-Jena-Leipzig, Leipzig, Germany

**Keywords:** Transcription factor binding sites, De novo motif discovery, Intra-motif dependencies, Model selection, ChIP-seq data

## Abstract

**Background:**

Statistical modeling of transcription factor binding sites is one of the classical fields in bioinformatics. The position weight matrix (PWM) model, which assumes statistical independence among all nucleotides in a binding site, used to be the standard model for this task for more than three decades but its simple assumptions are increasingly put into question. Recent high-throughput sequencing methods have provided data sets of sufficient size and quality for studying the benefits of more complex models. However, learning more complex models typically entails the danger of overfitting, and while model classes that dynamically adapt the model complexity to data have been developed, effective model selection is to date only possible for fully observable data, but not, e.g., within de novo motif discovery.

**Results:**

To address this issue, we propose a stochastic algorithm for performing robust model selection in a latent variable setting. This algorithm yields a solution without relying on hyperparameter-tuning via massive cross-validation or other computationally expensive resampling techniques. Using this algorithm for learning inhomogeneous parsimonious Markov models, we study the degree of putative higher-order intra-motif dependencies for transcription factor binding sites inferred via de novo motif discovery from ChIP-seq data. We find that intra-motif dependencies are prevalent and not limited to first-order dependencies among directly adjacent nucleotides, but that second-order models appear to be the significantly better choice.

**Conclusions:**

The traditional PWM model appears to be indeed insufficient to infer realistic sequence motifs, as it is on average outperformed by more complex models that take into account intra-motif dependencies. Moreover, using such models together with an appropriate model selection procedure does not lead to a significant performance loss in comparison with the PWM model for any of the studied transcription factors. Hence, we find it worthwhile to recommend that any modern motif discovery algorithm should attempt to take into account intra-motif dependencies.

**Electronic supplementary material:**

The online version of this article (doi:10.1186/s12859-015-0797-4) contains supplementary material, which is available to authorized users.

## Background

Statistical modeling of functional DNA sequences such as transcription factor binding sites is one of the classical fields in bioinformatics. The statistical representation of binding sites of the same transcription factor (TF), which can be understood as a probability distribution over all possible DNA sequences of a certain length, is typically called *sequence motif*. There are at least two different computational concepts for inferring such a sequence motif from data, namely (i) from a given set of aligned training sequences of fixed length [1–7], which may have been extracted from databases such as JASPAR [8] or TRANSFAC [9], and (ii) from a set of non-aligned sequences of arbitrary length, such as promoters of genes that are regulated by the TF of interest, that are assumed to contain a binding site, but where its precise location is not known. The latter task is typically called *de novo motif discovery*, and a plethora of research has been dedicated to this non-trivial problem [10–18].

The most common statistical model for modeling transcription factor binding sites, which most of the aforementioned methods are based on [19], is the so called position weight matrix (PWM) model [1, 2]. The features taken into account by a PWM model correspond to those that can be graphically represented by a sequence logo [20]. The PWM model assumes statistical independence among all nucleotides in the motif and thus corresponds to the biophysical assumption that binding affinities of nucleotides within a DNA binding site to the corresponding DNA-binding protein are additive [21]. Due to this independence assumption, the PWM model requires comparatively few parameters that can be robustly estimated even from few and noisy training sequences, but there is an ongoing discussion about its capability of accurately modeling protein-DNA interaction [21–27]. With the rise of high-throughput techniques such as ChIP-seq [28], the size and quality of available training data sets increases, which in turn makes the use of more complex models promising.

Examples for alternative statistical models of higher complexity are the weight array model [3], Bayesian trees [4], and the generalized weight matrix model [29], all of which take into account first-order dependencies. Utilizing first-order dependencies for modeling and predicting transcription factor binding sites has been the focus of recent research [17, 18, 30–32].

However, there is no clear justification why putative intra-motif dependencies should be limited to directly adjacent nucleotides or nucleotide pairs within the motif. Markov models of higher order, permuted Markov models [7] or Bayesian networks [4, 33] are all capable of taking into account intra-motif dependencies beyond nucleotide pairs, but share the problem that the number of model parameters grows exponentially with the model order. A large number of model parameters in relation to sample size inevitably leads to the problem of overfitting, i.e., adapting the model parameters to noise in the training data instead of capturing only relevant features that are generalizable to previously unseen test data. While nowadays larger data sets could allow more parameters than needed to specify a PWM, overshooting with respect to the model complexity, i.e., number of model parameters, remains a non-negligible risk and has prevented the utilization of higher-order dependencies so far.

Recently, inhomogeneous parsimonious Markov models (PMMs) [34] have been proposed with the aim of allowing a fine-grained position-specific adaption of model complexity based on observed data. PMMs contain as special case the simple PWM model [1, 2], the weight array model [3], traditional inhomogeneous Markov models of higher order, and variable order Markov models [6], but their structural flexibility reaches further. PMMs allow for taking into account higher-order dependencies while using only a few more parameters than the PWM model, which is achieved by restricting the space of conditional probability parameters at a certain position to these coinciding with a parsimonious context tree (PCT) [35].

**Fig. 1:**
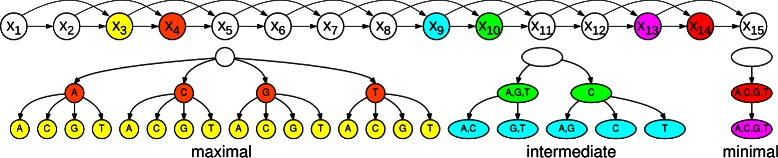
Inhomogeneous Parsimonious Markov model of order two for a motif of width 15. The nucleotide distribution of each position in the sequence may depend on the dinucleotide at the two previous positions. Parsimonious context trees (PCTs) are here used for reducing the parameter space by merging context sequences to sets of sequences, interpolating between traditional Markov model (maximal PCT) and the PWM model (minimal PCT). Exemplary PCTs, which cover both special cases and one intermediate case are shown for position 5, position 11, and position 15. The nodes in these PCTs are colored according to the conditioning random variables they correspond to

Besides the fine-grained adaption of model complexity to data, PMMs also allow us to adapt the model complexity in a position-specific manner, so that the degree of dependency that is taken into account may vary along the motif due to allowing a different PCT for each position (Fig. 1). Hence PMMs allow for interpolating between traditional Markov models of fixed order, which are obtained when all PCTs in the model are maximal, and the PWM model, which is obtained when all PCTs in the model are minimal. The presence of PCTs has the consequence that learning PMMs does not only consist of estimating conditional probability parameters, but also involves a structure learning (or model selection) step, which attempts to find a PCT that optimally reflects statistical dependencies present in the data for each position in the motif.

Applied within de novo motif discovery from ChIP-seq data, PMMs have been successfully utilized to unveil higher-order dependencies within the sequence motif recognized by the human insulator-binding protein CTCF [36]. The learning approach for PMM-based de novo motif discovery, which is based on maximizing the posterior of the model using the expectation-maximization (EM) algorithm, allows a smooth adaption of model complexity to data. However, maximizing the posterior has the disadvantage that the learned model complexity critically depends on the value of one external hyperparameter used in the structure prior and intuitively choosing a reasonable value for this hyperparameter is difficult.

As a result, learning PMMs within de novo motif discovery is to date only possible by hyperparameter-tuning via massive cross-validation on the training data, which requires restarting the EM algorithm for different hyperparameter values, different cross-validation iterations, and different initial parameter values required in any case for coping with local optima. Let us consider the realistic example that a single run of an EM algorithm requires one hour to converge and that we choose ten independent restarts with different initial parameter values to reduce the probability of converging to low local optima. Even if we want to test only 30 different hyperparameter values and perform only a ten-fold cross-validation for evaluating each of them, the required running time would be 3×10^3^ CPU hours compared to only 10 CPU hours required without hyper parameter tuning. This immense computational effort for PMM-based motif discovery using the previously proposed learning approach renders the analysis of a large number of data sets virtually impossible.

In this work, we conduct such a large-scale study, which aims at investigating the presence or absence of intra-motif dependencies of putative higher order and their utility for de novo motif discovery based on ChIP-seq data for a variety of different transcription factors. For this purpose, we propose a robust learning approach for PMMs in the presence of latent variables (‘Methods’), which avoids the specification of hyperparameters and thus does not require hyperparameter-tuning. Using this algorithm for de novo motif discovery, we are capable of robustly inferring models of different order, reflecting a different degree of putative intra-motif dependency, from on ChIP-seq data. Based on the performance of these models for the task of classifying previously unseen test data, we may reason about the presence or absence of statistical dependencies of a certain degree.

The rest of this manuscript is organized as follows. We present the main results from the motif discovery on ChIP-seq data in the next section, which consist of a quantitative comparison of different models based on classification results and a qualitative analysis of identified intra-motif dependencies. In the following ‘Discussion’ section, we summarize the key observations and elaborate on the lessons learned. We define the model, specify the robust learning algorithm, and describe the origin of the analyzed data in the ‘Methods’ section at the end of the manuscript.

## Results

In this work, we investigate 50 different human ChIP-seq data sets from the Uniform TFBS track of the ENCODE project [37, 38] (‘Methods’). First, we infer an overrepresented sequence motif in each of these data sets by using models that take into account different degrees of intra-motif dependencies within motif discovery and we discuss several obstacles that arise due to the nature of ChIP-seq data. We then evaluate the classification performance of the underlying models on previously unseen test data and investigate how widespread intra-motif dependencies in putative binding sites of human transcription factors are. Finally, we conduct a qualitative analysis of the resulting sequence motifs for several transcription factors in order to investigate statistical features that enable an improved motif discovery.

### Preliminary data analysis

A ChIP-seq experiment produces a set of sequences, each of which has a binding affinity of possible different strength to a particular DNA binding protein of interest, which is typically a transcription factor (TF). However, this does not necessarily imply that there is a specific binding motif for this TF within these sequences. There are cases in which a TF is only indirectly associated with the DNA molecule, e.g., due to binding to other DNA-binding proteins. Moreover, there are basal TFs that bind rather unspecifically but are required to form the transcription initiation complex. In these cases, we may observe a ChIP-seq data set that does not contain one clearly overrepresented sequence motif, and including such a data set into a systematic evaluation of intra-motif dependencies could and often would yield misleading results.

For that reason, we perform in a first study for all 50 data sets a robust motif discovery (‘Methods’) with the aim of investigating the predicted binding sites, before comparing classification performances in the next section. In all cases, we attempt to find a motif of width *W*=20. Even though not all TFs can be expected to bind to such a long binding site, adding some possibly uninformative positions is less harmful than not being able to take into account all informative positions. As a motif model we use inhomogeneous parsimonious Markov models [34] of order 0–4, which also includes the standard PWM model [1, 2] that is equivalent to the PMM of order zero.

We predict binding sites in the positive sequences based on the motif models learned during motif discovery and the negative data for determining the significance level (‘Methods’). We investigate the sequence logos to quantify the mononucleotide distribution of these predicted binding sites (see Additional file 1).

**Fig. 2:**
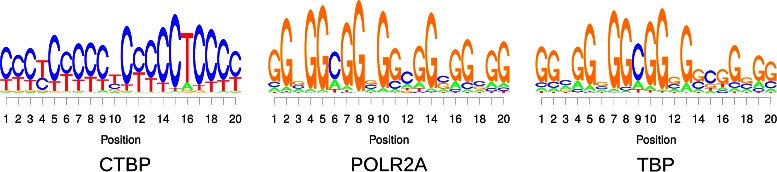
Sequence logos of data sets without meaningful motif. In some cases, we find these repetitive structures that can hardly be considered as transcription factor binding sites

In 14 of 50 cases we observe that the motif discovery consistently identifies only repetitive structures, but no sequence motifs in the traditional sense. Obvious examples for such data sets are shown in Fig. 2. While there may be transcription factors that do bind a long repetitive structure, the identification of a repeat is also a typical result obtained when the data set contains no specific motif, which could be expected for ChIP-experiments against POLR2A or TBP indeed. Hence, we exclude these 14 data sets from all following analyses and focus on the remaining 36 data sets for which the algorithm identifies distinct sequence motifs.

**Fig. 3:**
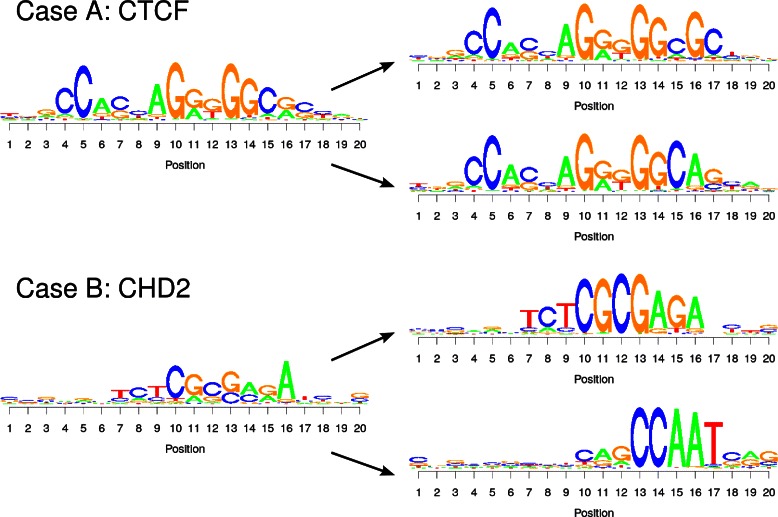
Intra-motif dependencies and multiple motif occurrences. The two sequence logos of the left show the motif inferred by a PMM1 for the CTCF and CHD2 data sets. After applying a mixture model of two PWM components on the underlying predicted binding sites, we obtain a clustering that can be represented by two sequence logos. For CTCF, we observe that both sequence logos are similar and resemble the original prediction, and differences among both logos are just an alternative representation of the dependencies at the 3’ end of the motif. For CHD2, we observe that both sequence logos are fundamentally different at all positions. Hence, the corresponding binding sites appear to be bound by two different proteins and just co-occur within the same ChIP-seq data set

Here, we still face the obstacle that in some cases attempting to take into account intra-motif dependencies might cause a dependency model to infer at least two different sequence motifs from one data set and to combine them into a single model. In order to test for this situation, we learn from all PMM-predicted sets of binding sites a mixture model of two PWM model components (‘Methods’). Investigating the resulting sequence logos, we can distinguish two cases, which are illustrated in Fig. 3.

For some data sets, we observe that the two sequence logos after de-mixing are in accordance with the sequence logo from the initial set of predicted binding sites. In these cases it is a plausible assumption that all binding sites are the target of the same protein, and differences in the sequence logos after de-mixing are just a reflection of the dependencies in the data. Hence, a dependency model is learning correctly a sophisticated representation of the binding motif of one transcription factor. One prominent example is CTCF, where it is known that strong dependencies at the 3’ end exist [36].

For other data sets, however, we observe two fundamentally different sequence logos after de-mixing, which also disagree with the sequence logo from the initial prediction, which is actually a mixture of both. Here, we may speculate that we observe binding sites of at least two different proteins in the same ChIP-seq data set, which may happen if two transcription factors act for a substantial number of target genes in concert and bind to DNA predominantly in close proximity. Using a dependency model might thus also lead to learn the representation of more than one motif into one statistical model. This may be beneficial from statistical point of view for increasing the likelihood of the model, but it may not be an appropriate reflection of biological reality. One example is the CHD2 data set, which contains both a TCTCGCGAGA and a CCAAT consensus, which appear to be targets of two different proteins or otherwise overrepresented nucleotide sequences.

In the following investigation of intra-motif dependencies we intend to distinguish these two cases. We thus quantify the difference between two sequence logos after de-mixing by computation of the Jensen-Shannon divergence [39] (JSD) among both underlying PWMs (‘Methods’). For each data set, we compute the JSD for all four PMM-based predictions (order 1 to order 4), and average the resulting JSD values (see Additional file 2). We finally consider all data sets with an average JSD below a threshold of 0.18 as containing a motif of a single TF only, and refer to them as Category A in the following. We further consider all data sets with an average JSD above this threshold as putatively contaminated by the presence of multiple motifs, and refer to them as Category B.

### Classification results

In the previous section, we observed that two types of the 36 data sets that contain at least one prevalent motif can and should be qualitatively distinguished. Now we conduct a quantitative analysis of the presence of intra-motif dependence for all these data sets. To this end, we perform a *fragment-based classification* (‘Methods’), which is a method for assessing the quality of different motif models within the same general motif discovery framework when no ground truth w.r.t. to binding site locations is known. For the purpose of robust evaluation, we perform a ten-fold cross-validation. We use the area under the ROC curve (AUC) averaged over the ten cross-validation iterations as performance measure.

**Fig. 4:**
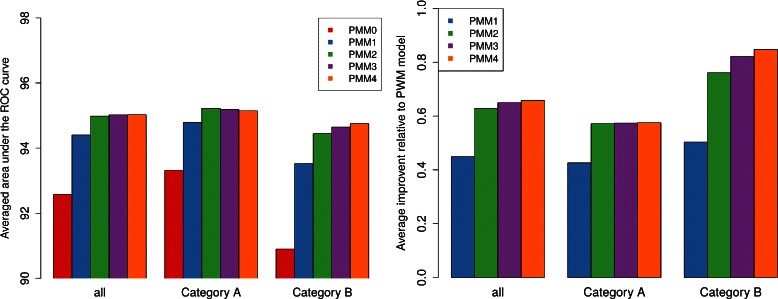
Aggregated results of fragment-based classification. The left figure shows the AUC for different models in percent averaged over (i) all ten cross-validation iterations for each data set as well as (ii) over all data sets and subgroups thereof. Right figure shows the relative improvement of PMMs of different order in relation to the PWM model according to the *Ψ*_*d*_ as defined in Eq. 1, which is also averaged (i) over all cross-validation iterations for each data set as well as (ii) over all data sets and subgroups thereof

First, we plot the averaged AUC values over all data sets, and separated among both categories A and B (Fig. 4, left plot). Generally, all four PMMs of orders 1 to 4 yield substantially higher averaged AUC values than the PWM model, which indicates that intra-motif dependencies are prevalent and that neglecting them yields a substantially decreased classification capability of the inferred motif. Moreover we find that second-order models are for all data groups on average better than first-order models. Higher-order models yield on average not a substantial further improvement, but there is also no substantial decrease in classification performance, from which we may conclude that the method is fairly robust against the choice of maximal model order.

Analyzing the difference between category A and B, we find that the magnitude of improvement differs among both categories, though the general conclusions are not overly affected. We observe that the PWM model yields a worse classification performance for category B than for category A, which is not surprising as in the presence of multiple motifs the PWM model can use only one of them for classification. Conversely, the room for improvement by allowing dependencies is higher for category B, and we even observe a slight performance increase towards third- and fourth-order models.

In order to quantify the statistical significance or non-significance of the observed differences among different model orders, we apply the Wilcoxon signed-rank test [40], which is a paired difference test, to the population of AUC values resulting from the classification of two different models. All *p*-values for comparing models with the null hypothesis of their mean AUC values being identical are shown in Additional file 3. Summarizing the results by using *α*=0.01, we confirm that for all groups the improvement of all models towards the PWM is highly significant, and also the increase from first to second order passes the significance test. The difference among higher-order models (second to fourth order) is not significant when considering the average over all data sets, though.

Averaging AUC values over different data sets has one drawback, namely AUC values are not directly comparable, as the classification problem is different for each data set. Different ChIP-seq data sets are of different size w.r.t. both number of peaks of sufficient quality and length of individual sequences. Moreover, the putative motif may be of different length and information content. Averaging over all AUC values for one model might yield a bias towards data sets that yield comparatively low AUC values. For that reason, it would be beneficial to devise a method that allows fair comparison of models, yet treats all data sets on equal ground.

Since we use a fragment-based classification for comparing different models from which we reason about the presence or absence of intra-motif dependencies, we are mainly interested in the relative difference of a PMM of a certain order to the PWM model. Let AUC_0_ denote the area under the ROC curve for the PWM model (a PMM of order 0), and AUC_*d*_ denote the area under the area ROC curve for the PMM of order *d*. We compute

(1) $$ \Psi_{d} = \log_{2} \left(\frac{1-\text{AUC}_{0}}{1-\text{AUC}_{d}} \right),  $$

which measures the improvement of the PMM of order *d* in relation to the PWM model.

The idea behind this score to compute the difference to a perfect classification for both approaches, with subsequently computing their ratio. The binary logarithm is then taken to ensure that positive values indicate an improvement of the PMM of order *d* over the PWM model, and negative values indicate the opposite. Obtaining *Ψ*_*d*_=1 thus indicates that using a PMM of order *d* reduces, in relation to the PWM model, the distance to a perfect classification by a factor of 2.

We compute *Ψ*_*d*_ for *d*=1,…,4 for all data sets, and we display the averaged results (Fig. 4, right plot). We observe the results to be generally in accordance with the previous findings, but the effects become a bit more pronounced.

**Fig. 5:**
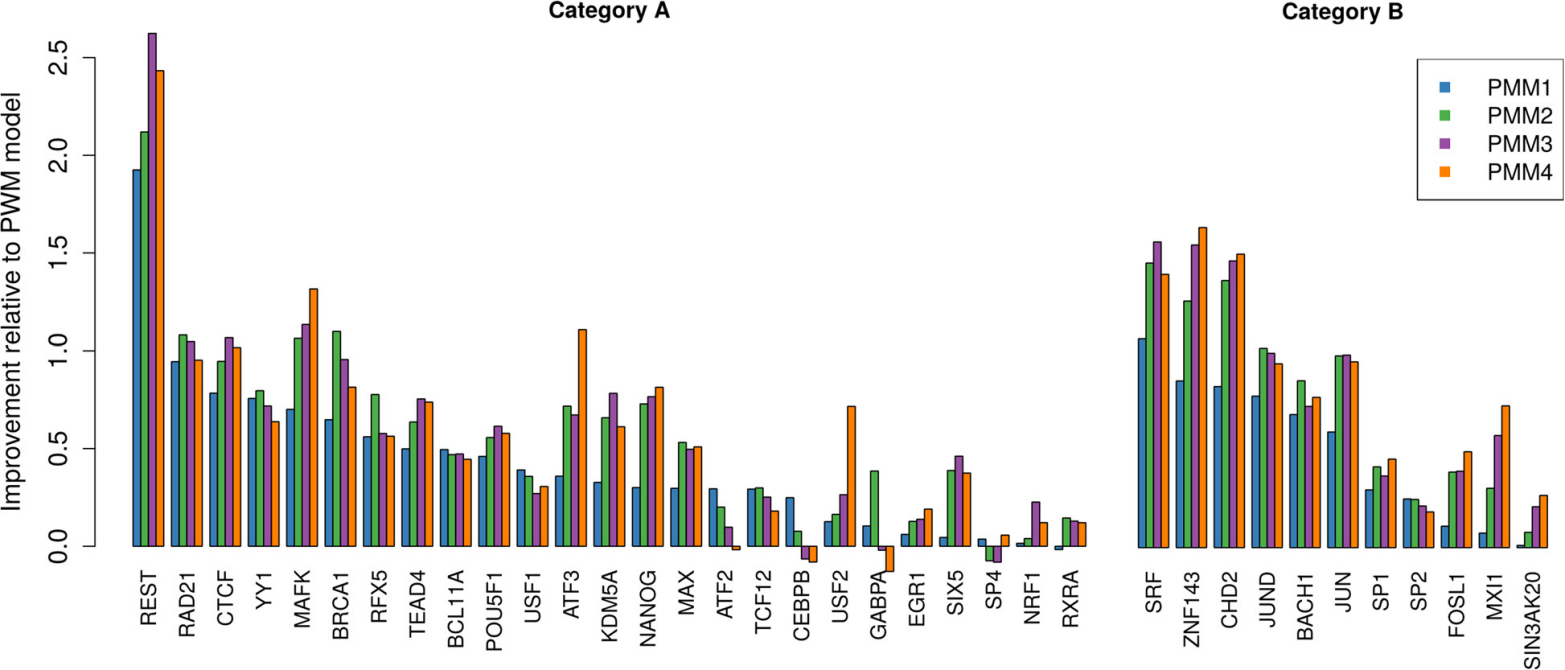
Data set specific improvements. We show *Ψ*_*d*_ for PMMs of different order for all data sets that contain at least one motif, each averaged over the ten cross-validation iterations. For the vast majority of data sets, we find that taking into account intra-motif dependencies via PMMs improves motif discovery substantially

Now we take a more fine-grained view and investigate these improvements separately for all data sets. In Fig. 5, we plot *Ψ*_*d*_ for all data sets individually. The results are ordered by *Ψ*_1_, i.e., the effect on classification performance from taking into account only first-order dependencies.

In order to test for statistical significance of one particular model order in relation to another, we perform for each data set a Wilcoxon signed rank test based on the ten cross-validation iterations. We display the summarized results, hereby distinguishing category A and B, in Table 1.

**Table 1:** Data set specific significance test

	PWM	PMM1	PMM2	PMM3	PMM4
PWM	–	0+0	0+0	0+0	0+0
PMM1	15+6	–	0+0	1+0	2+0
PMM2	15+8	4+6	–	0+0	2+0
PMM3	14+9	6+4	1+1	–	0+0
PMM4	16+9	7+5	3+3	2+0	–

For each data and every combination of models, we perform a Wilcoxon signed rank test (*α*=0.01) comparing the distribution of AUC values from the different cross-validation iterations. The entry in *a*+*b* the *i*-th row and the *j*-th column denotes the number of data sets for which the model corresponding to row *i* yields a significantly better classification performance than the model corresponding to column *j*, where *a* denotes the number of significant differences in category A data sets, and *b* the number of significant differences in category *b* data sets. We find many instances where increasing the model order yields to significantly better classification (lower triangle), but only few instances where it yields a significantly worse classification (upper triangle)

We observe in Fig. 5 that using first-order dependencies improves classification performance for almost all data sets. We find in 15 of 25 cases for category A and in 6 of 9 cases for category B that this improvement is indeed statistically significant (Table 1). Conversely, there is not a single case in which a PWM model would have been the significantly better choice. Hence, taking into account first-order intra-motif dependencies is always at least as good as using a PWM model and in the majority of cases it yields a substantial improvement.

In some cases, modeling first-order dependencies is the best choice, as higher-order models do not yield an improved classification performance or even a slightly decreased classification performance. However, there are also many cases in which PMMs of higher order improve classification. In several cases, such BRCA1, increasing the model order to two improves classification, but further increasing it has only little effect, from which we may conclude that here there are no substantial intra-motif dependencies beyond order two. In case of REST, increasing model order up to three significantly improves classification, whereas attempting to take into account fourth-order dependencies is not beneficial anymore. Finally, there are a few cases where utilizing dependencies up to order four gradually improves classification, and significant examples are here USF2 and ATF3.

Interestingly, category B data sets do not show a substantially larger amount of higher-order dependencies, from which we may conclude that a motif mixture can be equally well represented by first- or at least second-order models. The slight, even though non-significant, improvement towards third- and fourth-order models observed for category B, which is missing in category A (Fig. 4), can be explained by an opposite effect. For category A, we identify two cases (GABPA and ATF2) in which attempting to take into account third- and fourth-order dependencies actually yields to a significantly decreased classification performance in relation to first- and second-order models, which also lowers the average statistics, even though the magnitude of the performance loss is fairly small.

### Qualitative dependency analysis

Finally, we analyze dependencies of different order for some transcription factors of category A, which are not a putative mixture of motifs of two different transcription factors, on a qualitative level. To this end, we utilize the models inferred via motif discovery from the entire training data sets, which we obtained for the data set categorization.

**Fig. 6:**
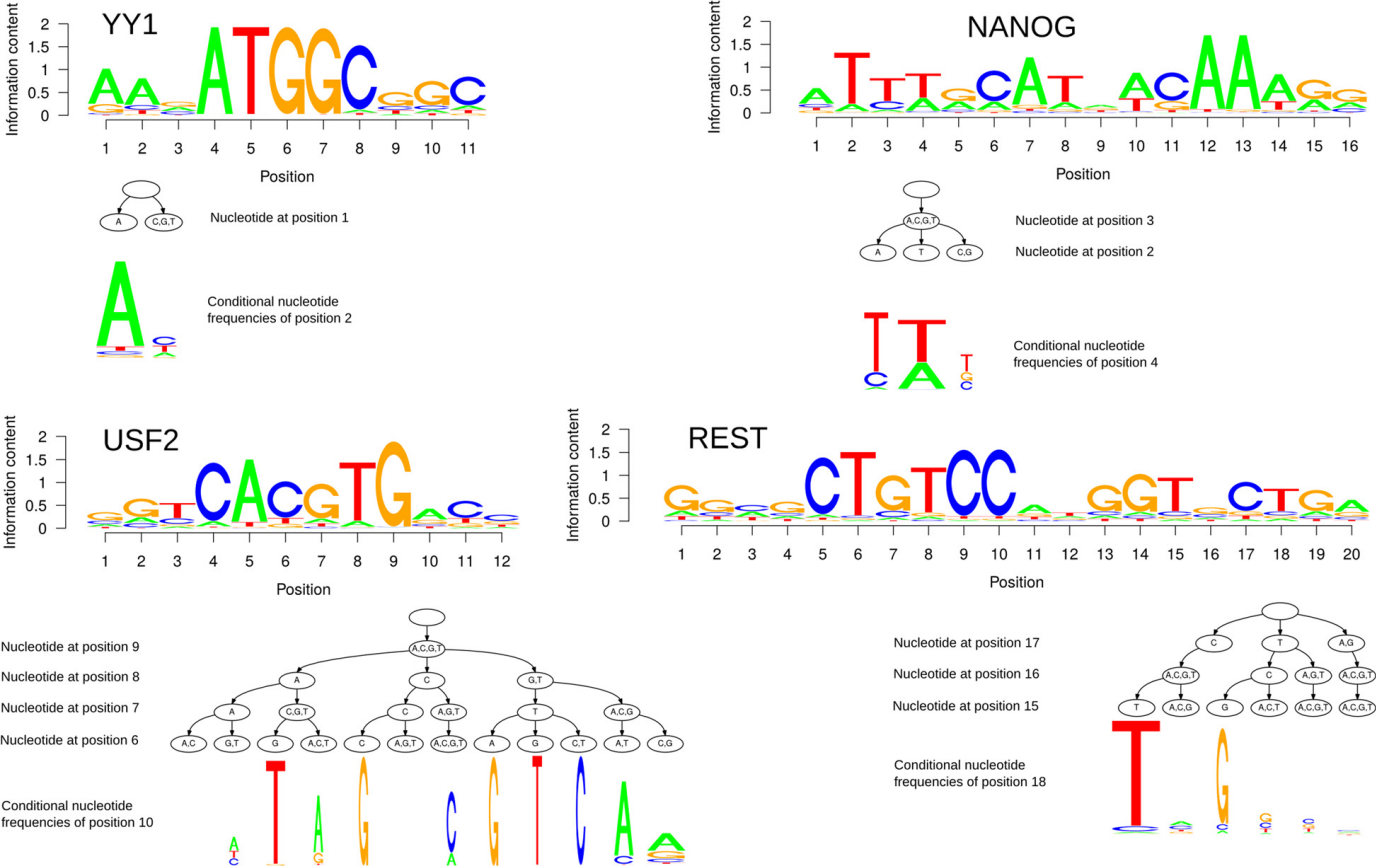
Sequence logos and position-specific dependency refinements of several transcription factors. We visualize dependencies of order 1–4 for YY1, NANOG, REST, and USF2 by plotting the traditional sequence logo for each TF and show a position-specific refinement by showing the PCT at one position together with the conditional sequence logos of each leaf in the PCT. The width of the conditional sequence logo is scaled according to the number of sequences in the data that match the particular context, with broad nucleotide stacks representing frequent and narrow nucleotide stacks representing infrequent contexts

In Fig. 6, we plot the sequence logos of the predicted binding sites for four different TFs (YY1, NANOG, REST, and USF2), each being an example of one particular maximal order (one to four) of intra-motif dependency. We display for each of the four TFs one position-specific refinement visualized by a conditional sequence logo (‘Methods’).

For the transcription factor YY1, we display the result of the first-order PMM, which is significantly better than a PWM model and not significantly worse than any model of higher order. We find at position 2 in particular a strong dependence to position 1. Observing an A at the first position yields a high probability of observing another A at the second position, whereas for all other observations in the context the probability distribution is near to uniform. This essentially reveals that the YY1-motif has an optional AA-dinucleotide at the 5’-end, which is present in many but not all binding sites.

In case of NANOG, we display the result of the second-order PMM, which is here significantly better than a PWM model and a first-order PMM, but not significantly worse than a model of higher order. We find a pronounced dependency at position 4 to position 2, skipping position 3. Here, the consensus nucleotide is identical for all conditional distributions, but both the information content and the second most frequent nucleotides vary from context to context.

For REST, we display the result of the third-order PMM, which is a significantly better motif representation than lower-order models and performs not significantly worse than a fourth-order PMM. We find a clear third-order dependency at position 18 to the previous three positions. Here, it differentiates between observing either T, when having observed TNC as previous trinucleotide, or G when having observed GCT as previous trinucleotide. The remaining contexts show a widely uniform distribution of nucleotides. This dependency is similar to YY1, as it indicates that the nucleotides at the 3’ are not be present in all binding sites, but are somewhat optional. However, if occurring at all, then GGT at position 13–15 and CTG at position 17–19 appear predominantly together.

USF2 is one of the few cases where a fourth-order model is significantly better than all lower-order models. We thus display the result of the fourth-order PMM, and find a fourth-order dependence at position 10. This example shows that a seemingly uninformative position may become informative when considering the context, as observing an A or T at position 6 can increase the probability of observing an A at position 10.

These examples demonstrate that taking into account position-specific intra-motif dependencies of different orders do not only improve motif discovery and classification performance, but that they can also be qualitatively identified and visualized.

## Discussion

We have investigated the prevalence of intra-motif dependencies in transcription factor binding sites as well as the task of utilizing them for improving de novo motif discovery from ChIP-seq data. However, we have observed in the previous sections that a certain caution in drawing conclusions from such studies is warranted. In several cases, an improved classification of ChIP-positives to background can be caused by the presence of multiple motifs in the data, which may be explained more appropriately by two independent sequence motifs instead of one motif with weak consensus but high dependencies. Being aware of this problem, we devised a threshold-based method for distinguishing multi-motif occurrences from “true” intra-motif dependencies.

It should be noted that some cases are at the borderline and no threshold can give a perfect separation between both cases. For instance, the data sets JUN and JUND yield a high Jensen-Shannon divergence because roughly half of the binding sites show a deletion of single nucleotide in the motif center. Such a feature yields strong dependencies and also a high evidence for multiple motifs, even though we still may consider all binding sites to be bound by the same protein.

But even for those datasets that appear to contain predominantly binding sites corresponding to one motif of a single TF, we find that de novo motif discovery can be substantially improved by modeling intra-motif dependencies. Taking into account dependencies with PMMs does not only significantly outperform the PWM model on average, but there is not a single case where using a PWM model would have been a significantly better choice than any PMM, all of which are capable of adapting their model structure to data. These findings indicate that intra-motif dependencies are widely present and not an exception that only occurs for special TFs such as CTCF [36].

An interesting question concerns the biophysical cause of the observed intra-motif dependencies. One hypothesis is that transcription factors mainly detect the shape of the DNA and not precise nucleotides [17]. On the other hand, we have also observed a case like YY1 where a dependency actually reveals the presence or absence of a specific dinucleotide that is present in some, but not all, binding sites. Since YY1 is a zinc-finger protein, this feature might be explained by a somewhat optional contact of one of the zinc fingers to DNA, as proposed for another zinc finger protein [41]. Hence, there may be not one single, but rather multiple biophysical reasons for observing intra-motif dependencies, but in all cases we are capable of exploiting these additional statistical features to obtain a more realistic representation of binding sites in terms of a more accurate statistical model.

We must be cautious with extreme conclusions and stop short of declaring the ultimate death of the PWM model. For estimating a sequence motif from limited training data, e.g., a few experimentally verified binding sites, the PWM model will certainly remain the optimal choice. For fully observable data, more than 10^2^ training sequences are typically required before any model with more parameters than a PWM model can effectively utilize additional information in the data [34, 42]. Moreover, due to its small parameter space, the PWM model is more robust to low-quality data, where models that attempt to infer dependencies may be more prone to adapting to noise. For inferring sequence motifs from high quality ChIP-seq data, however, neglecting intra-motif dependencies yields – on average – a significantly worse motif, so modern motif discovery algorithms should attempt to take into account intra-motif dependencies to some degree.

In addition to the general presence of intra-motif dependencies, we have investigated their degree by comparing different models that are capable of taking into account dependencies up to a certain maximal order. We find that for about a third of the relevant data sets intra-motif dependencies of higher order yield an improved classification performance and thus an improved de novo motif discovery. The degree of dependency that continues to increase classification varies among data sets. We find the second-order PMM to be the best choice on average, closely followed the third- and fourth-order PMMs with a non-significant difference. There are noteworthy cases, though, in which third- and fourth-order models are helpful for enhancing motif discovery, but this kind of higher-order dependency appears to be the exception.

It may be possible that the data quality resulting from current ChIP-seq experiments is just not high enough to effectively utilize all intra-motif dependencies that protein-DNA binding truly involves. Whereas a decade ago data quality prohibited effectively utilizing dependencies at all, it is nowadays apparently possible to reliably take into account first- and second-order dependencies when present in data and to choose for a simple model otherwise. We may thus speculate that advances in experimental technologies will enable to effectively learn even more sophisticated models that are capable of robustly taking into account additional features.

De novo motif discovery remains interesting in the sense that it is not well understood what exactly a sequence motif truly is. For more than two decades, the sequence logo, which corresponds to the sufficient statistics or to the parameters of the PWM model, used to be the common perception of a sequence motif of a TF. While this notion is gradually shifting towards first-order dependency models, the results from this work indicate that at least second-order dependencies should be considered, and we believe that this is not the end of the story. Perhaps it may be more appropriate to generally define a sequence motif as the set of all statistical features describing the set of binding sites of the TF of interest. Attempting to unveil which statistical features are actually relevant for the process of protein-DNA binding and which are not will certainly remain an ambitious goal for years to come.

## Conclusions

In this work, we have studied intra-motif dependencies within transcription factor binding sites based on ChIP-seq data. Due to the danger of overfitting, such a study requires (i) a model that is capable of adapting its complexity dynamically to a given ChIP-seq data set and (ii) an effective learning algorithm that allows a model selection even within de novo motif discovery, i.e., in the presence of latent variables. Both is given by inhomogeneous parsimonious Markov models and a stochastic algorithm for model selection in the presence of latent variables as presented in this work. In the empirical part, we have seen that a certain caution in interpreting results is warranted, as ChIP-seq data sets can contain secondary motifs that can lead to an overestimation of motif complexity. We have corrected against this effect and observed that intra-motif dependencies remain prevalent and that attempting to model second-order dependencies appears to be the best choice on average. Hence, we suggest that any modern motif discovery algorithm should attempt to take into account additional statistical features beyond position-specific mononucleotide frequencies to some degree.

## Methods

In the first two sections, we discuss the model and the robust learning approach that allows PMM model selection without relying on hyperparameter-tuning via cross-validation. Afterwards, we describe the procedures for predicting binding sites from learned motif models, the test for multiple motif occurrence, and the classification approach used for comparing different models. Finally, we provide information regarding the implementation and the data sets used in the study.

### Model specification

The input data consist of a set of *N* ChIP-seq positive sequences of possibly different lengths $\vec {L}=(L_{1},\dots,L_{N})$. We denote the *ℓ*-th nucleotide in the *i*-th input sequence by $x_{i,\ell } \in \mathcal {A} = \{\texttt {A,C,G,T}\}$, the *i*-th sequence by $\vec {x}_{i} = (x_{1},\ldots,x_{{L}_{i}})$, and the complete data set by $\mathbf {x}= (\vec {x}_{1},\ldots,\vec {x}_{N})$.

For modeling the occurrence of binding sites in such ChIP-seq sequences, we use the *one occurrence per sequence* (OOPS) model [43], which is a popular choice for modeling occurrences of binding sites in DNA sequences, and corresponds, despite its simple assumption of allowing only a single occurrence of one motif type, well to the data a ChIP-seq experiment is supposed to generate. It consists of an arbitrary *motif model* of width *W*, parameterized by *Θ*_m_, and an arbitrary *flanking model*, parameterized by *Θ*_f_. Both are combined into the full parameter set **Θ**=(*Θ*_m_,*Θ*_f_). The OOPS model makes use of two latent variables per sequence, with *v*_*i*_∈{1,…,*L*_*i*_−*W*+1} denoting the binding site position in the *i*-th sequence and *s*_*i*_∈{F,R} denoting the strand orientation of the binding site that may be located either on the forward (F) or on the reverse complementary (R) strand. We combine the latent variables of the complete data set by $\vec {{v}}=({v}_{1},\dots,{v}_{{N}})$ and $\vec {s}=({s}_{1},\dots,{s}_{{N}})$.

The conditional likelihood of a sequence ${\vec {x}}_{i}$ given latent variables *v*_*i*_ and *s*_*i*_ and parameters **Θ** is thus given by

$$ \begin{aligned} P({\vec{x}}_{i}|{v}_{i},{s}_{i},{\mathbf{\Theta}}) &= P_{\text{\small{f}}}({x}_{i,1},\dots,{x}_{i,{v}_{i}-1}|{\Theta}_{\text{\small{f}}})\\ & \quad\cdot P_{\text{\small{f}}}({x}_{i,{v}_{i}+{W}},\dots,{x}_{i,{L}_{i}}|{\Theta}_{\text{\small{f}}})\\ & \quad\cdot P_{\text{\small{m}}}({x}_{i,{v}_{i}},\dots,{x}_{i,{v}_{i}+{W} -1}|{\Theta}_{\text{\small{m}}})^{\delta_{{s}_{i},\text{\tiny F}}}\\ &\quad\cdot P_{\text{\small{m}} }(\text{rc}({x}_{i,{v}_{i}},\dots,{x}_{i,{v}_{i}+{W} -1})|{\Theta}_{\text{\small{m}} })^{\delta_{{s}_{i},\text{\tiny R}}}, \end{aligned}  $$

where $\text {rc}({\vec {x}})$ returns the reverse complement of ${\vec {x}} $ and *δ*_*a,b*_ denotes the Kronecker delta, which returns 1 if *a*=*b* and 0 otherwise. For the entire data set ${\mathbf {x} =({\vec {x}}_{1},\dots,{\vec {x}}_{N})}$, we assume statistical independence among individual sequences, hence

(2) $$\begin{array}{@{}rcl@{}} P({\mathbf{x}} |\vec{{v}},\vec{s},\mathbf{\Theta}) = \prod\limits_{i=1}^{N} P\left(\vec{x}_{i}| v_{i},s_{i},\Theta_{\text{\small{m}}},\Theta_{\text{\small{f}}}\right). \end{array} $$

As flanking model we use a homogeneous Markov model, and *Θ*_f_ comprises the corresponding conditional probability parameters. For the case studies in this work, we use a second-order model, which is typically sufficient to capture abundant repetitive structures, such as mono- and dinucleotide repeats, that may hamper the discovery of a functional motif.

As motif model, we use an inhomogeneous parsimonious Markov model (PMM) [34], which is a model that makes a position specific use of parsimonious context trees [35] (PCTs) for taking into account statistical dependencies while keeping the parameter space small. A PCT of depth *d* is a rooted, balanced tree that represents all *context sequences* of length *d* and organizes them in different groups, represented by the leaves in the tree. Each node of a PCT is labeled by a non-empty subset of $\mathcal {A}$, except for the root, which is labeled by the empty subset. The set of labels of all children of an arbitrary inner node forms a partition of $\mathcal {A}$. It follows that the cross product of the symbol sets encountered along each path from a leaf to the root defines a non-empty subset of $\mathcal {A}^{d}$, which we call *context*. Hence, a context is a set of context sequences, and the set of the contexts of all leaves of a PCT forms a partition of $\mathcal {A}^{d}$.

A typical PCTs of depth two is shown in Fig. 1. It represents the organization of all 16 possible context sequences of length two into 5 contexts, namely {AA,AG,AT,CA,CG,CT}, {GA,GG,GT,TA,TG,TT}, {AC,GC}, {CC}, and {TC}.

The idea of a PMM is to spend one conditional probability parameter vector for each context, i.e., for each group of context sequences defined by the PCT, whereas a traditional Markov model spends one conditional probability parameter vector for each context sequence. Whereas the parameter space of traditional Markov models grows exponentially with the length of the context sequences, i.e., the degree of dependency that is to be taken into account, this does not necessarily occur for parsimonious Markov models. Hence, PMMs allow for a fine-grained tradeoff between modeling dependencies and keeping the parameter space as small as possible, which attempts to avoid overfitting.

Let **x** denote here a set of binding sites of fixed width *W*. An inhomogeneous PMM contains exactly *W* PCTs $\vec {\tau }=(\tau _{1},\ldots,\tau _{W})$. We denote a single context, i.e., a group of context sequences, by *c*, and the conditional probability of observing a symbol $a \in \mathcal {A}$ given that the concatenation of the preceding *d* symbols is in *c* by *θ*_*ca*_. We denote the model parameters of one PCT at a single position by $\left (\tau, (\vec {{\theta }}_{{c}})_{{c}\in \tau }\right)$ and all parameters of the motif model by

$$\begin{array}{@{}rcl@{}} \Theta_{\text{\small{m}}}= \left(\tau_{\ell}, (\vec{\theta}_{\ell c})_{{c}\in\tau_{\ell}}\right)_{\ell \in (1,\ldots,{W})}. \end{array} $$

The likelihood of a PMM is then given by

(3) $$\begin{array}{@{}rcl@{}} P({\mathbf{x}} |{\Theta}_{\text{\small{m}} }) = \prod\limits_{\ell=1}^{{W}} \prod\limits_{{c}\in \tau_{\ell}} \prod\limits_{{a}\in {\mathcal{A}}} \left({\theta}_{\ell {c} {a}}\right)^{{N}_{\ell {c} {a}}}, \end{array} $$

where *N*_*ℓca*_ is the number of occurrences of symbol *a* at position *ℓ* in all sequences of **x** where the concatenation of the symbols from position *ℓ*−*d* to position *ℓ*−1 is in *c*.

PMMs are a quite general model class that contains both fixed-order Markov models and variable-order Markov models [6] as special cases, which can both be obtained by restricting the structural flexibility of the PCTs [44]. It should be noted that the *order of a PMM* always pertains to the maximal order, i.e., to the maximal depth of the parsimonious context trees and thus the maximal number of context positions that can be taken into account. It can happen in the course of model selection that a learned PCT neglects some positions in the context completely, which is a desired feature and in fact the very reason why a PMM of higher order can be expected to perform always at least as good as a PMM of lower order on the same data.

### Robust learning approach

Learning PMMs for de novo motif discovery has been previously conducted by specifying a prior distribution to the OOPS model and all of its components and applying the maximum a posteriori (MAP) principle [36]. This maximization problem cannot be solved analytically, but the posterior can be monotonically increased using a modified EM algorithm [45], which requires an M step that performs model selection according to the MAP principle. Since asymptotically the prior is dominated by the data, model selection according to the MAP principle is not consistent, i.e., it is asymptotically equivalent to model selection according to the maximum likelihood principle, which yields – in the case of nested models – always the largest model structure with the highest dimensionality of the parameter space. Hence, the EM algorithm is very sensitive to the choice of the structure prior, which needs to counterbalance growing sample size. As a result, tuning of the prior hyperparameter on the training data, e.g., by internal cross-validation, is essential for conducting any reasonable model selection within the EM algorithm.

Here, we propose a robust alternative to learning PMMs within motif discovery that avoids the specification of hyperparameters, and thus also avoids hyperparameter-tuning, based on the following two key ideas. First, we phrase the entire motif discovery problem primarily as model selection problem for the motif model, whereas the previous approach [36] attempts to optimize the posterior of the entire latent variable model. Second, we reduce this model selection problem, which still involves latent variables, to the simple case of fully observable data.

In such a case, when all binding sites are already known and pre-aligned, i.e., experimentally confirmed binding sites or data sets from a database such as JASPAR [8], the task of learning PMMs involves no latent variables at all and can thus be carried out exactly. While the first approach for learning PMMs from fully observable data was still based on a Bayesian approach with massive hyperparameter-tuning [34], it could be later shown that this effort might not be necessary [42]. The combination of the *Bayesian information criterion (BIC)* [46] as structure score for finding the optimal PCT structures and the *factorized sequential Normalized Maximum Likelihood (fsNML)* [47] for estimating the conditional probability parameters appears to constitute a robust alternative [42]. This method avoids the specification of hyperparameters and consequently does not require any hyperparameter-tuning, but it is nevertheless highly competitive w.r.t. predictive performance.

For de novo motif discovery, however, the setting is more complex as latent variables $(\vec {v},\vec {s})$ are involved, and it may not be immediately obvious how to benefit from the previous results for fully observable data. We define $\mathbf {x}_{\vec {v},\vec {s}}$ as the set of binding sites from **x** that are given by latent variable configuration $(\vec {v},\vec {s})$, and ${N}^{\vec {v},\vec {s}}_{{\ell }ca}$ as the corresponding counts of observing nucleotide *a* at position *ℓ* given context *c* at the previous positions. With latent variables given, we can thus compute the structure parameter estimate $\hat {\tau }_{\ell }$ as maximum of the BIC structure score

(4) $$  {}S_{\text{\tiny BIC}}(\tau_{\ell}|{\mathbf{x}}_{\vec{{v} },\vec{{s}}}) \!= \!\sum\limits_{{c} \in \tau_{\ell}} {{N}^{\vec{{v}},\vec{{s}}}_{\ell {c} {a}}} \log \left(\frac{{N}^{\vec{{v}},\vec{{s}}}_{\ell {c} {a} }}{{N}^{\vec{{v}},\vec{{s}}}_{\ell {c} \cdot}}\right) -\frac{1}{2}|\tau_{\ell}|(|{\mathcal{A}} |-1)\log({N}).  $$

BIC has the form of a penalized log-likelihood, where the penalty term serves as a regularization. It consists of the number of free parameters of the current model, which are given by $|\tau _{\ell }|(|{\mathcal {A}} |-1)$ and the sample size *N* of the training data, thus involving no additional hyperparameters that need to be optimized. The penalization of BIC is known to be rather strong and it favors comparatively sparse model structures with few parameters. For learning PMMs this behavior has been empirically shown to be actually beneficial [42] as it allows a robust model selection that calls for additional parameters only if they are clearly supported by dependencies observed in the data but chooses for simplicity otherwise.

With given model structure $\hat {\tau }_{\ell }$, we fix the corresponding conditional probability parameters according to the fsNML parameter estimate

(5) $$\begin{array}{@{}rcl@{}} \hat{\theta}_{{\ell}{ca}}^{\text{\tiny fsNML}}({\mathbf{x}}_{\vec{{v}},\vec{{s}}}) = \frac{e({N}^{\vec{{v}},\vec{{s} }}_{\ell {c} {a} })({N}^{\vec{{v} },\vec{{s} }}_{\ell {c} {a}}+1)}{\sum_{b\in{\mathcal{A}} }e({N}^{\vec{{v} },\vec{{s} }}_{\ell {c} b})({N}^{\vec{{v} },\vec{{s} }}_{\ell {c} b}+1)}, \end{array} $$

where $e(N) = (\frac {N+1}{N})^{N}$ for *N*>0 and *e*(0)=1.

In de novo motif discovery, however, the realizations of the latent variables are not known. Since the main goal is selecting PCTs of adequate complexity, we also phrase the de novo motif discovery problem as model selection problem, namely finding the optimal model structure for a putative sequence motif in the data. In other words, we seek the latent variable assignment that yields the highest structure score, and according to the OOPS model assumption, we thus seek

(6) $$  (\vec{{v}}^{\star},\vec{{s}}^{\star}) = \mathop{\text{argmax}}\limits_{(\vec{{v} },\vec{{s}})} \max\limits_{\vec{\tau}} \sum\limits_{\ell=1}^{{W}} S_{\text{\tiny BIC}}(\tau_{\ell}|{\mathbf{x}}_{\vec{{v}},\vec{{s}}}).  $$

Unfortunately, this optimization problem cannot be solved exactly without the explicit enumeration of all possible realizations $(\vec {{v}},\vec {{s}})$. This obstacle is common among almost all learning tasks involving latent variables, and, as it is common in such cases, we rely also here on an approximate solution. After having obtained such approximation for $(\vec {{v}}^{\star },\vec {{s}}^{\star })$, however, we can compute the optimal PCT structures and conditional probability parameters exactly according Eqs. 4 and 5.

We approximate Eq. 6 by an iterative stochastic algorithm shown in Algorithm 1 that can be perceived as a variant of a stochastic EM algorithm [48]. It relies on sampling a latent variable configuration based on data and current model parameters, a step that is related to the Gibbs motif sampler [10]. After having sampled a latent variable configuration in a loop over all *N* input sequences, a second loop over all *W* motif positions involves completely deterministic steps. PCTs are learned according to BIC structure score using the DP algorithm for exact PCT maximization [35], and conditional probability parameters for all leaves of all PCTs are estimated according to fsNML.



This algorithm generates for each of *R* restarts a series of $(\vec {{v}},\vec {{s}})^{({t})}$ for *t*=1,…,*T*. We run the algorithm at least *T* iteration steps, but require in addition that we did not observe an improvement of the score during the last *T* ^′^ iteration steps before termination.

Using that strategy, we are capable of limiting the running time of the algorithm in most cases, while still ensuring that a reasonably stable score and thus motif is found. For all case studies in this work, we use *T*=50, *T* ^′^=10, and *R*=10.

### Binding site prediction and analysis

Based on the learned parameters $\hat {{\mathbf {\Theta }} }$, we compute the likelihood

(7) $$ {}P({\vec{x}}_{i},{v}_{i}\,=\,j|\hat{{\mathbf{\Theta}}})\! =\! P({\vec{x}}_{i},{v}_{i}\,=\,j,{s}_{i}\,=\,\mathrm{F}|\hat{{\mathbf{\Theta}}}) + P({\vec{x}}_{i},{v}_{i}\,=\,j,{s}_{i}\,=\,\mathrm{R}|\hat{{\mathbf{\Theta}}})  $$

for each possible start position *j* in each sequence ${\vec {x}}_{i}$ in the negative data. Obtaining a list of likelihood values, we compute the empirical probability distribution of this list, and determine a threshold *Z* as the likelihood corresponding to the lowest (10^−4^)-quantile, which we use to predict all subsequences of width *W* in each sequence ${\vec {x}}_{i}$ beginning at position *j* in the positive data set satisfying

(8) $$ P({\vec{x}}_{i},{v}_{i}=j|\hat{{\mathbf{\Theta}}}) > {Z}  $$

as binding sites. We compute sequence logos [20] from the mononucleotide counts of the predicted binding sites. In addition, we also compute conditional sequence logos [34], which are representations of the conditional nucleotide frequencies for all contexts represented by a PCT at a single position in a binding site. The conditional nucleotide frequencies of a context are visualized as nucleotide stack comparable to a single position in a traditional sequence logo. However, since not all contexts are equally important, the width of the nucleotide stacks of a conditional sequence logo is scaled according to the number of sequences this particular context represents. Scaling is done according to three categories: For a dominant context that represents more than 50 % of all possible sequences in the data, the nucleotide stack obtains a standard width. For a normal context that represents between 5 % to 50 % of the sequences, the width of the corresponding nucleotide stack is reduced by a factor of 2, whereas for rare contexts that represent less than 5 % of sequences in the data, the width is reduced by a factor of 4.

### Test for multiple motif occurrence

In order to test to which degree a set of predicted binding sites might be also explained by being bound by two different proteins, we use the following strategy. First, we cluster a set of binding sites predicted with a particular method from a particular data sets using a two-component mixture of PWM models using Jstacs [49]. Let $\vec {p}_{j}^{(1)}$ and $\vec {p}_{j}^{(2)}$ denote the *j*-th PWM column of both clusters, and let *w*_1_ and *w*_2_ denote here the weights of each cluster, i.e., the relative number of sequences contained in it. The *j*-th column of the PWM of the original, unclustered binding sites is thus $\vec {p}_{j}=w_{1}p_{j}^{(1)}+w_{2}p_{j}^{(2)}$. Next, we compute the Jensen-Shannon divergence [39] of the *j*-th column by

(9) $$ \text{JSD}_{j}\left(\vec{p}_{j}^{(1)},\vec{p}_{j}^{(2}\right)= w_{1} \text{KLD}\left(\vec{p}_{j}^{(1)}|\vec{p}\right)+w_{2} \text{KLD}\left(\vec{p}_{j}^{(2)}|\vec{p}\right),  $$

where KLD denotes the Kullback-Leibler divergence between two probability distributions, which is defined by

(10) $$ \text{KLD}(\vec{p},\vec{q}) = \sum\limits_{i} p_{i}\ln \frac{p(i)}{q(i)}.  $$

As aggregated JSD for the entire motif we use the sum of the JSD values over all positions, i.e., $\text {JSD} = \sum _{j=1}^{{W} } \text {JSD}_{j}$. Finally, we average the JSDs obtained from prediction of PMMs of order one to four. Repeating this procedure for each data set, we are capable of quantifying for which TFs multiple motifs within a ChIP-seq data set may be the true reason for strong intra-motif dependencies observed. In this work, we use a threshold of 0.18 and categorize all data sets with a smaller JSD as single motif occurrence, and all data sets with a larger JSD as multiple motif occurrence.

### Fragment-based classification

Evaluate different models for de novo motif discovery is not straightforward, as there is typically no ground truth with respect to true binding sites available. We thus use an evaluation of different motif models solely based on ChIP-seq positive and negative data [36], where the classification problem is to classify long sequence fragments into these that contain an instance of the motif, and are thus ChIP-seq positives, and those that do not, thus being ChIP-seq negatives. We call this indirect approach of evaluating different motif models *fragment-based classification*.

For the sake of robustness, we perform a ten-fold cross-validation, so we divide training and test data sets in ten subsets each. We use each subset once as test data set, while the union of the remaining nine data sets constitutes the training set of the particular cross-validation iteration.

For each iteration, we estimate the parameters of the flanking model *Θ*_f_ from the union of the positive and the negative data set, and afterwards perform a de novo motif discovery on the positive data set in order to estimate *Θ*_m_. For all methods under consideration, we build a classifier that consists of an OOPS model for class one and a second-order homogeneous Markov model for class two, where the parameters of the homogeneous Markov model are identical to *Θ*_f_.

We classify all sequences in the test data sets and compute the area under the ROC curve (AUC) given the true class labels. Hence, any improvement in AUC is a result of a more accurately estimated sequence motif, so the classification performance can be interpreted as an accuracy measure for the de novo motif discovery given a particular motif model.

### Implementation

All models and learning algorithms are implemented in Java and based on the Jstacs library [49]. Runnable JAR-files for model training, binding site prediction, and fragment-based classification are available on the project website [50].

### ChIP-seq data sets

We use all data sets that are available for the H1-hESC cell line from the Uniform TFBS track of the ENCODE project [37, 38], which comprises 50 different transcription factors. These data sets consists of a list of ChIP-seq peaks, identified by chromosome, start position, end position, and an enrichment score, which indicates the strength of the binding.

For each TF, we pick the top 20 *%* of the available peaks and extract the corresponding sequences from the human genome in order to build a positive data set. Next, we extract for each positive sequence two negative sequences of the same length from randomly sampled locations on the same chromosome, provided they do not overlap with a positive sequence or contain ambiguous nucleotides, and compile them into a negative data set. All positive and negative data sets with the cross-validation splits used in this study are available in FASTA format on the project website [50].

## Additional files

Additional file 1
**Sequence logos of predicted binding sites.** The file contains sequence logos of predicted binding sites for all 50 data sets and all five model orders. (PDF 4147.2 kb)

Additional file 2
**Average JSD values.** The plot shows the JSD values of the de-mixing experiment for all 36 data sets that contain sequence motifs other than repeats. (PDF 4.95 kb)

Additional file 3
***p*** **-values of model comparison.** The tables show *p*-values of Wilcoxon signed ranked tests comparing the different models, one for each group of data sets. (PDF 26.3 kb)

## References

[1] Stormo GD, Schneider TD, Gold LM (1982). Characterization of translational initiation sites in E.coli. Nucleic Acids Res.

[2] Staden R (1984). Computer methods to locate signals in nucleic acid sequences. Nucleic Acids Res.

[3] Zhang MQ, Marr TG (1993). A weight array method for splicing signals analysis. Comput Appl Biosci.

[4] Barash Y, Elidan G, Friedman N, Kaplan T (2003). Modeling dependencies in protein-DNA binding sites. Proceedings of the Seventh Annual International Conference on Research in Computational Molecular Biology.

[5] Rahmann S, Müller T, Vingron M (2003). On the power of profiles for transcription factor binding site detection. Stat Appl Genet Molec Biol.

[6] Ben-Gal I, Shani A, Gohr A, Grau J, Arviv S, Shmilovici A (2005). Identification of transcription factor binding sites with variable-order Bayesian networks. Bioinformatics.

[7] Zhao X, Huang H, Speed TP (2005). Finding short DNA motifs using permuted Markov models. J Comp Biol.

[8] Sandelin A, Alkema W, Engström P, Wasserman WW, Lenhard B (2004). JASPAR: an open-access database for eukaryotic transcription factor binding profiles. Nucleic Acids Res.

[9] Matys V, Fricke E, Geffers R, Gößling E, Haubrock M, Hehl R (2003). TRANSFAC: transcriptional regulation, from patterns to profiles. Nucleic Acids Res.

[10] Lawrence CE, Altschul SF, Boguski MS, Liu JS, Neuwald AF, Wootton JC (1993). Detecting subtle sequence signals: A Gibbs sampling strategy for multiple alignment. Science.

[11] Bailey TL, Williams N, Misleh C, Li WW (2006). MEME: discovering and analyzing DNA and protein sequence motifs. Nucleic Acids Res.

[12] Pavesi G, Mauri G, Pesole G (2001). An algorithm for finding signals of unknown length in DNA. Bioinformatics.

[13] Thompson W, Rouchka EC, Lawrence CE (2003). Gibbs recursive sampler: finding transcription factor binding sites. Nucleic Acids Res.

[14] Kim NK, Tharakaraman K, Mariño-Ramírez L, Spouge JL (2008). Finding sequence motifs with Bayesian models incorporating positional information: an application to transcription factor binding sites. BMC Bioinf.

[15] Keilwagen J, Grau J, Paponov IA, Posch S, Strickert M, Grosse I (2011). De-novo discovery of differentially abundant transcription factor binding sites including their positional preference. PLoS Comput Biol.

[16] Bi Y, Kim H, Gupta R, Davuluri RV (2011). Tree-based position weight matrix approach to model transcription factor binding site profiles. PLOS ONE.

[17] Mathelier A, Wasserman WW (2013). The next generation of transcription factor binding site prediction. PLoS Comput Biol.

[18] Grau J, Posch S, Grosse I, Keilwagen J (2013). A general approach for discriminative de novo motif discovery from high-throughput data. Nucleic Acids Res.

[19] Tran NTL, Huang CH (2014). A survey of motif finding web tools for detecting binding site motifs in ChIP-seq data. Biol Direct..

[20] Schneider TD, Stephens RM (1990). Sequence logos: A new way to display consensus sequences. Nucleic Acids Res.

[21] Benos PV, Bulyk M, Stormo GD (2002). Additivity in protein-DNA interactions: how good an approximation is it?. Nucleic Acids Res.

[22] O’Flanagan RA, Paillard G, Lavery R, Sengupta AM (2005). Non-additivity in protein-DNA binding. Bioinformatics.

[23] Badis G, Berger MF, Philippakis AA, Talukder S, Gehrke AR, Jaeger SA (2009). Diversity and complexity in DNA recognition by transcription factors. Science.

[24] Zhao Y, Stormo GD (2011). Quantitative analysis demonstrates most transcription factors require only simple models of specificity. Nat Biotechnol.

[25] Morris Q, Bulyk ML, Hughes TR (2011). Jury remains out on simple models of trancription factor specificity. Nat Biotechnol.

[26] Zhao Y, Ruan S, Pandey M, Stormo G (2012). Improved models for transcription factor binding site identification using nonindependent interactions. Genetics.

[27] Keilwagen J, Grau J (2015). Varying levels of complexity in transcription factor binding motifs. Nucleic Acids Res.

[28] Johnson DS, Mortazavi A, Myers RM, Wold B (2007). Genome-wide mapping of in vivo protein-DNA interactions. Science.

[29] Zhou Q, Liu JS (2004). Modeling with-motif dependence for transcription factor binding site prediction. Bioinformatics.

[30] Slattery M, Zhou T, Yang L, Dantas Machado AC, Gordan R, Rohs R (2014). Absence of a simple code: how transcription factors read the genome. Trends Biochem Sci.

[31] Yang L, Zhou T, Dror I, Mathelier A, Wasserman WW, Rohs R (2014). TFBSshape: a motif database for dna shape feature of transcription factor binding sites. Nucleic Acids Res.

[32] Siddharthan R (2010). Dinucleotide weight matrices for predicting transcription factor binding sites: Generalizing the position weight matrix. PLOS ONE.

[33] Heckerman G, Geiger D, Chickering D (1995). Learning Bayesian networks: The combination of knowledge and statistical data. Machine Learning.

[34] Eggeling R, Gohr A, Bourguignon PY, Wingender E, Grosse I (2013). Inhomogeneous parsimonious Markov models. Machine Learning and Knowledge Discovery in Databases - European Conference, ECML PKDD 2013, vol. 1.

[35] Bourguignon PY, Robelin D. Modèles de Markov parcimonieux: sélection de modele et estimation. In: Proceedings of JOBIM. Montréal: 2004.

[36] Eggeling R, Gohr A, Keilwagen J, Mohr M, Posch S, Smith AD (2014). On the value of intra-motif dependencies of human insulator protein CTCF. PLOS ONE.

[37] The ENCODE Project Consortium (2007). Identification and analysis of functional elements in 1 % of the human genome by the ENCODE pilot project. Nature.

[38] The ENCODE Project Consortium (2011). A user’s guide to the encyclopedia of DNA elements. PLoS Biol.

[39] Lin J (1991). Divergence measures based on the Shannon entropy. IEEE Trans Inform Theory.

[40] Wilcoxon F (1945). Individual comparisons by ranking methods. Biom Bull.

[41] Nakahashi H, Kwon KR, Resch W, Vian L, Dose M, Stavreva D (2013). A genome-wide map of CTCF multivalency redefines the CTCF code. Cell Rep.

[42] Eggeling R, Roos T, Myllymäki P, Grosse I. Robust learning of inhomogeneous PMMs. In: Proceedings of the 17th International Conference on Artificial Intelligence and Statistics (AISTATS). JMLR Workshop and Conference Proceedings, vol. 33: 2014. p. 229–237.

[43] Lawrence CE, Reilly AA (1990). An expectation maximization algorithm for the identification and characterization of common sites in unaligned biopolymer sequences. Proteins.

[44] Eggeling R, Koivisto M, Grosse I. Dealing with small data: On the generalization of context trees. In: Proceedings of the 32nd International Conference on Machine Learning (ICML). JMLR Workshop and Conference Proceedings, vol. 37: 2015.

[45] Dempster AP, Laird NM, Rubin DB (1977). Maximum likelihood from incomplete data via the EM algorithm. J R Stat Soc.

[46] Schwarz GE (1978). Estimating the dimension of a model. Ann Stat.

[47] Silander T, Roos T, Myllymäki P. Locally minimax optimal predictive modeling with Bayesian networks. In: Proceedings of the 12th International Conference on Artificial Intelligence and Statistics (AISTATS). JMLR Workshop and Conference Proceedings, vol. 5: 2009. p. 504–511.

[48] Nielsen SF (2000). The stochastic EM algorithm: Estimation and asymptotic results. Bernoulli.

[49] Grau J, Keilwagen J, Gohr A, Haldemann B, Posch S, Grosse I (2012). Jstacs: A Java framework for statistical analysis and classification of biological sequences. J Mach Learn Res.

[50] Eggeling R. Jstacs Project Website: PMMdeNovo. http://www.jstacs.de/index.php/PMMdeNovo. Accessed 16 June 2015.

