# Supplementary material for: Inferring intra-motif dependencies of DNA binding sites from ChIP-seq data
Source: BMC Bioinformatics. 2015 Nov 9;16:375. doi: 10.1186/s12859-015-0797-4 (PMC4640111; doi:10.1186/s12859-015-0797-4)

1 Evidence for (at least) one motif

ATF2 (1193 ChIP-Seq positives)

PWM **PMM1** **PMM2** **PMM3** **PMM4**

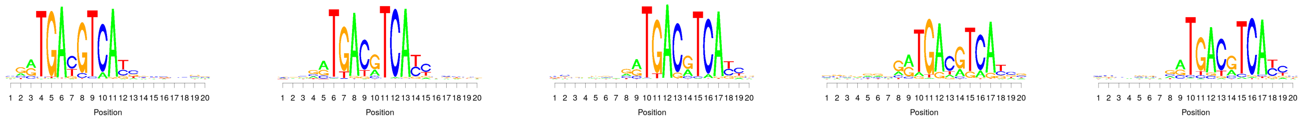

ATF3 (962 ChIP-Seq positives)

PWM **PMM1** **PMM2** **PMM3** **PMM4**

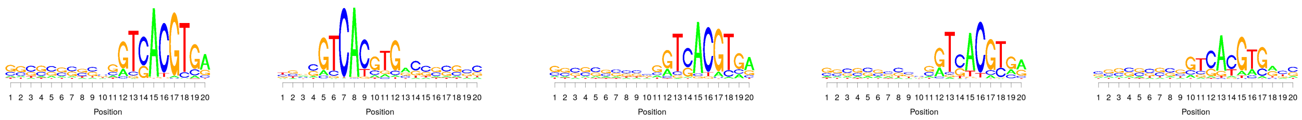

BACH1 (2292 ChIP-Seq positives)

PWM **PMM1** **PMM2** **PMM3** **PMM4**

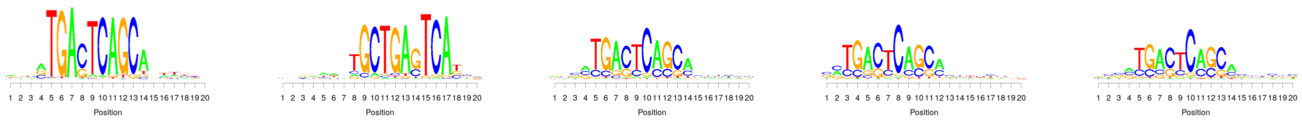

BCL11A (504 ChIP-Seq positives)

PWM **PMM1** **PMM2** **PMM3** **PMM4**

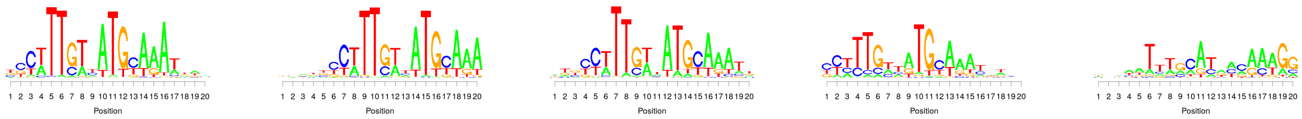

BRCA1 (405 ChIP-Seq positives)

PWM **PMM1** **PMM2** **PMM3** **PMM4**

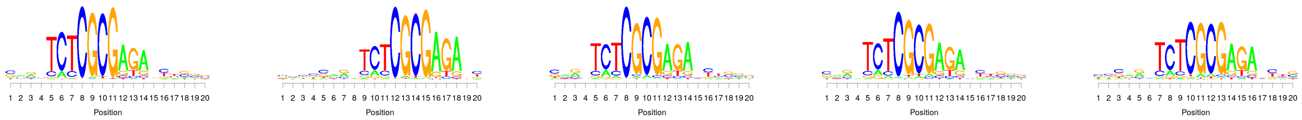

CEBPB (3112 ChIP-Seq positives)

PWM **PMM1** **PMM2** **PMM3** **PMM4**

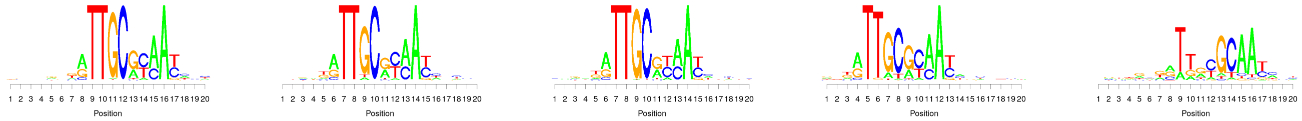

CHD2 (1368 ChIP-Seq positives)

PWM **PMM1** **PMM2** **PMM3** **PMM4**

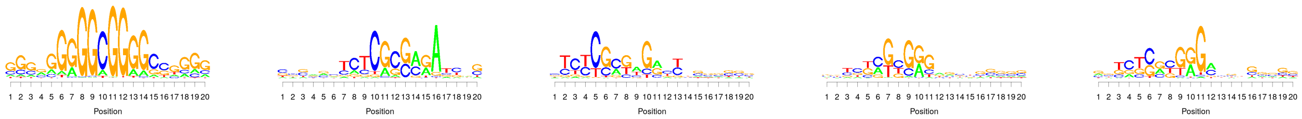

CTCF (10822 ChIP-Seq positives)

PWM **PMM1** **PMM2** **PMM3** **PMM4**

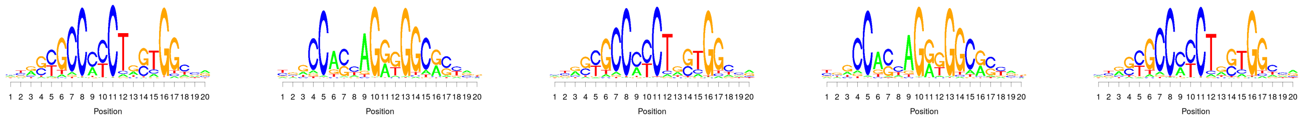

EGR1 (1749 ChIP-Seq positives)

PWM **PMM1** **PMM2** **PMM3** **PMM4**

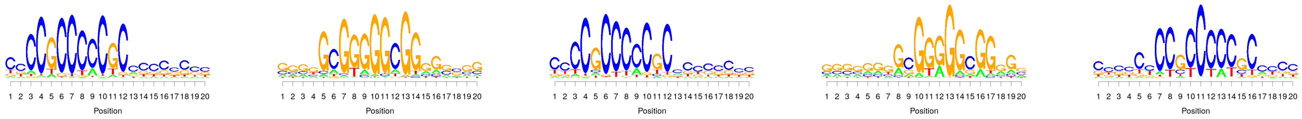

FOSL1 (223 ChIP-Seq positives)

PWM PMM1 PMM2 PMM3 PMM4

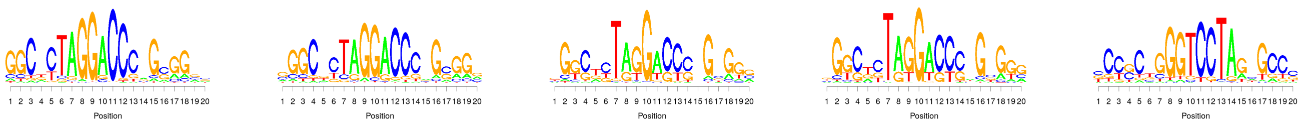

GABPA (1130 ChIP-Seq positives)

PWM PMM1 PMM2 PMM3 PMM4

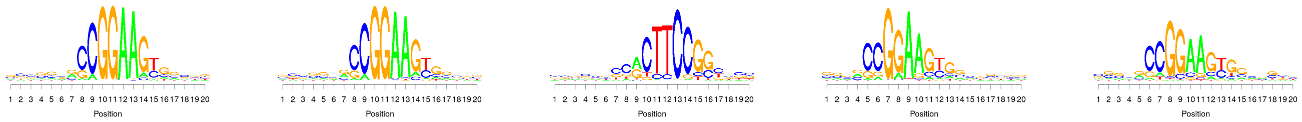

JUN (430 ChIP-Seq positives)

PWM PMM1 PMM2 PMM3 PMM4

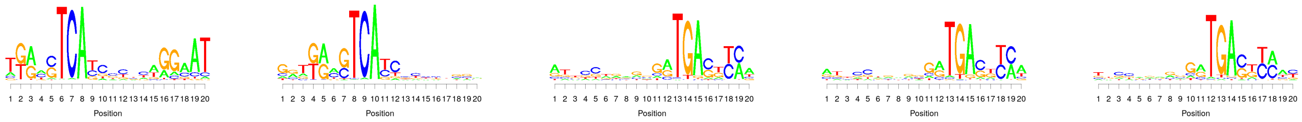

JUND (1690 ChIP-Seq positives)

PWM PMM1 PMM2 PMM3 PMM4

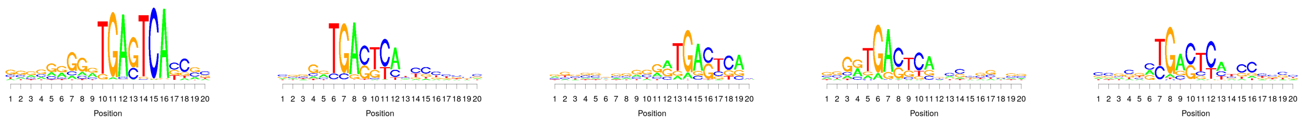

KDM5A (325 ChIP-Seq positives)

PWM PMM1 PMM2 PMM3 PMM4

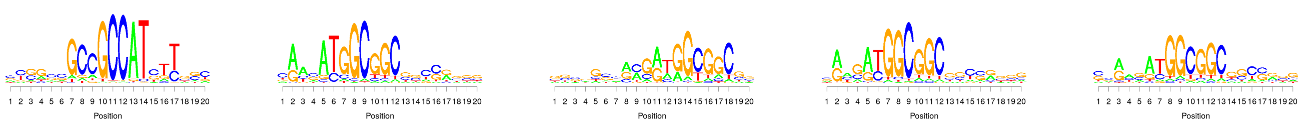

MAFK (2284 ChIP-Seq positives)

PWM	PMM1	PMM2	PMM3	PMM4

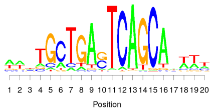

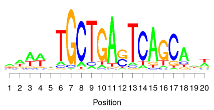

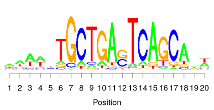

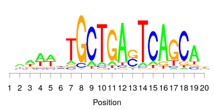

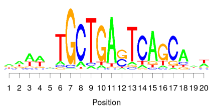

MAX (2225 ChIP-Seq positives)

PWM	PMM1	PMM2	PMM3	PMM4

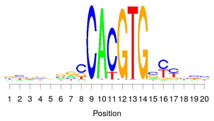

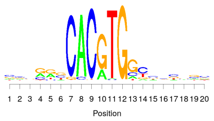

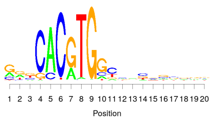

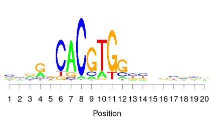

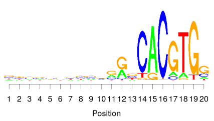

MXI1 (1271 ChIP-Seq positives)

PWM	PMM1	PMM2	PMM3	PMM4

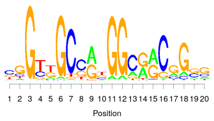

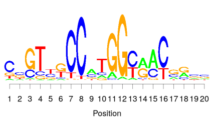

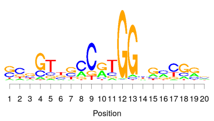

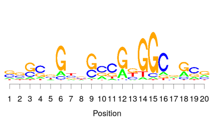

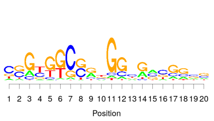

NANOG (1095 ChIP-Seq positives)

PWM	PMM1	PMM2	PMM3	PMM4

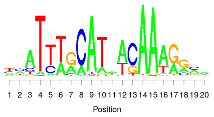

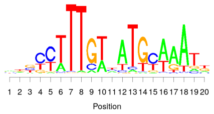

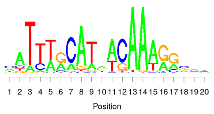

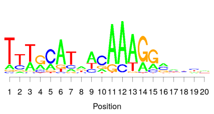

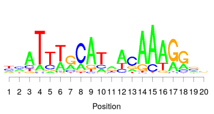

NRF1 (903 ChIP-Seq positives)

PWM	PMM1	PMM2	PMM3	PMM4

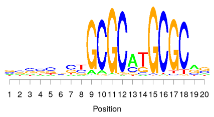

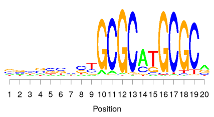

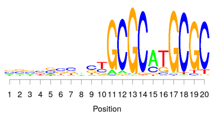

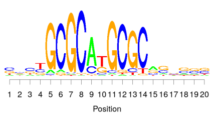

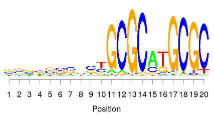

POU5F1 (800 ChIP-Seq positives)

PWM **PMM1** **PMM2** **PMM3** **PMM4**

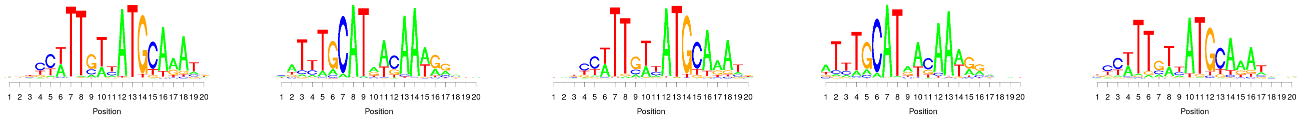

RAD21 (15136 ChIP-Seq positives)

PWM **PMM1** **PMM2** **PMM3** **PMM4**

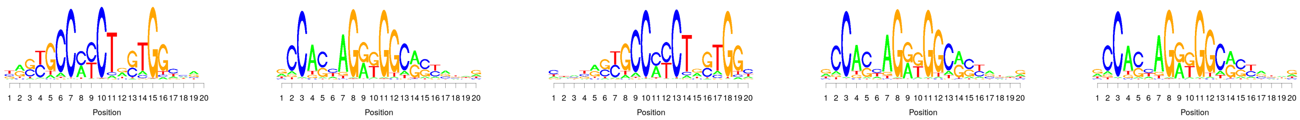

REST (2656 ChIP-Seq positives)

PWM **PMM1** **PMM2** **PMM3** **PMM4**

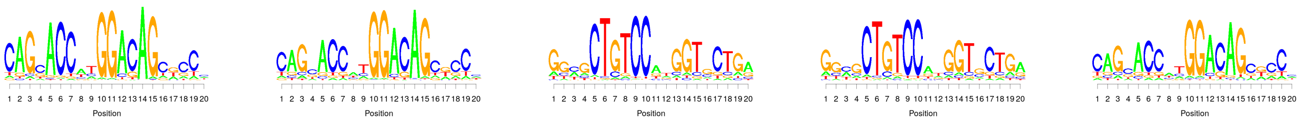

RFX5 (338 ChIP-Seq positives)

PWM **PMM1** **PMM2** **PMM3** **PMM4**

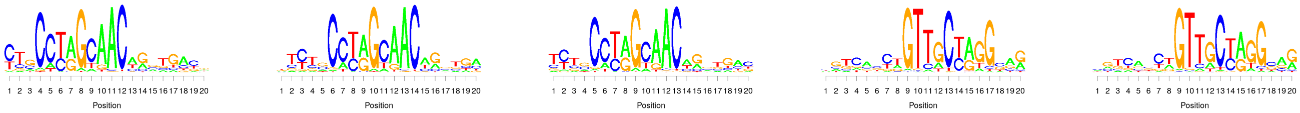

RXRA (262 ChIP-Seq positives)

PWM **PMM1** **PMM2** **PMM3** **PMM4**

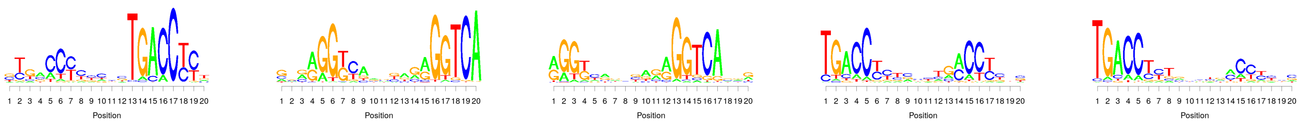

SIN3AK20 (4260 ChIP-Seq positives)

PWM	PMM1	PMM2	PMM3	PMM4

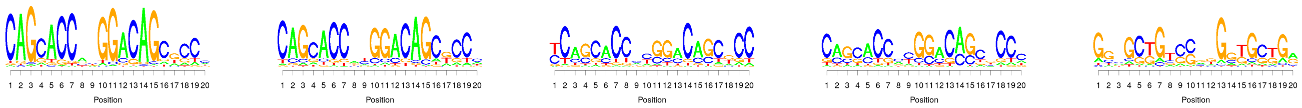

SIX5 (684 ChIP-Seq positives)

PWM	PMM1	PMM2	PMM3	PMM4

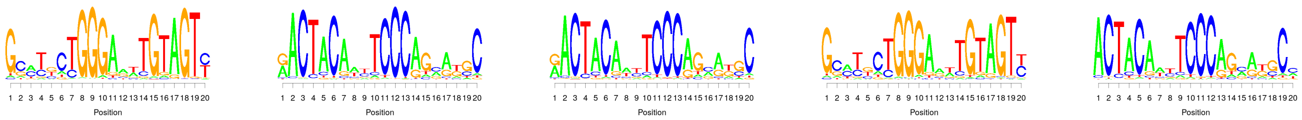

SP1 (3009 ChIP-Seq positives)

PWM	PMM1	PMM2	PMM3	PMM4

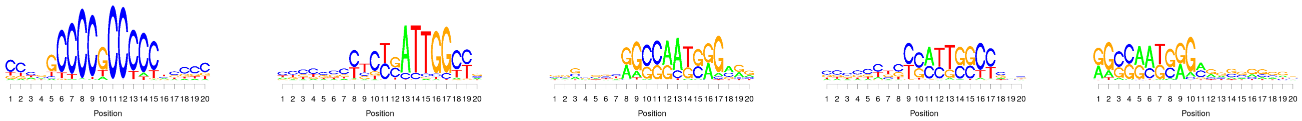

SP2 (492 ChIP-Seq positives)

PWM	PMM1	PMM2	PMM3	PMM4

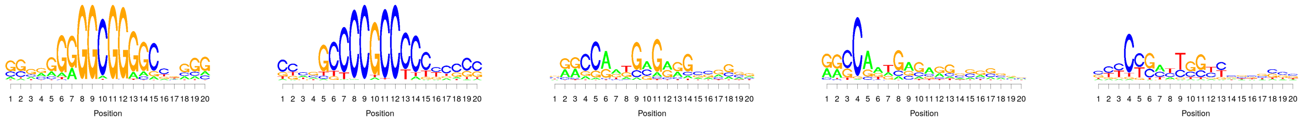

SP4 (1150 ChIP-Seq positives)

PWM	PMM1	PMM2	PMM3	PMM4

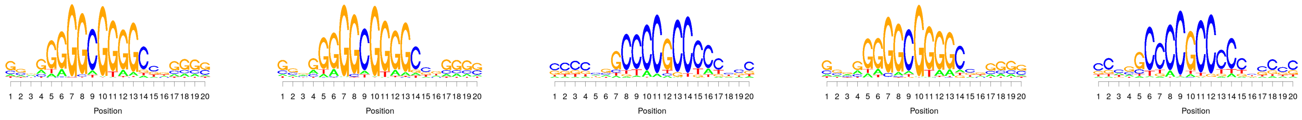

SRF (1020 ChIP-Seq positives)

PWM PMM1 PMM2 PMM3 PMM4

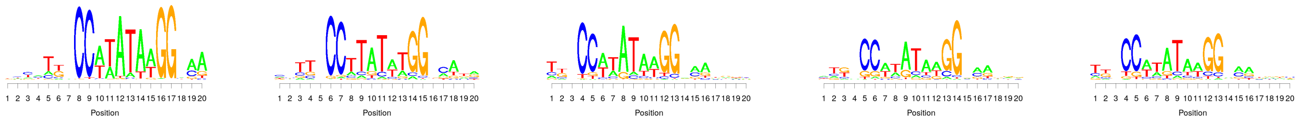

TCF12 (1567 ChIP-Seq positives)

PWM PMM1 PMM2 PMM3 PMM4

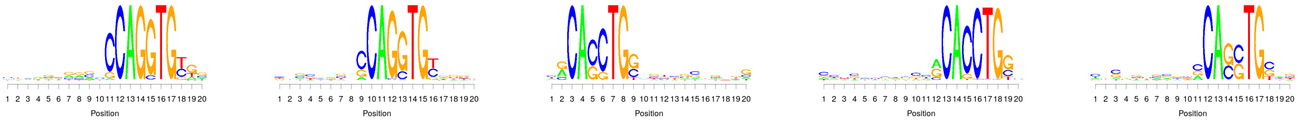

TEAD4 (3972 ChIP-Seq positives)

PWM PMM1 PMM2 PMM3 PMM4

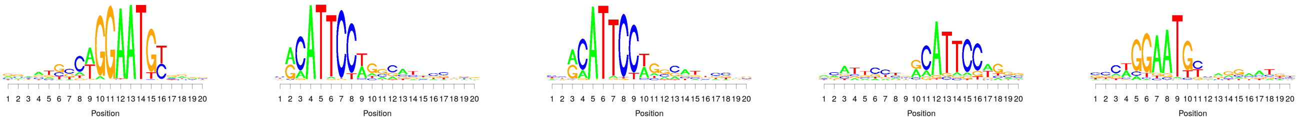

USF1 (5208 ChIP-Seq positives)

PWM PMM1 PMM2 PMM3 PMM4

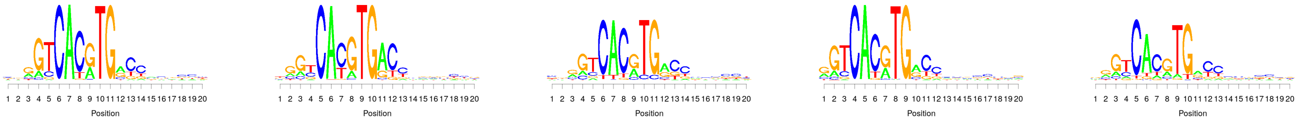

USF2 (1391 ChIP-Seq positives)

PWM PMM1 PMM2 PMM3 PMM4

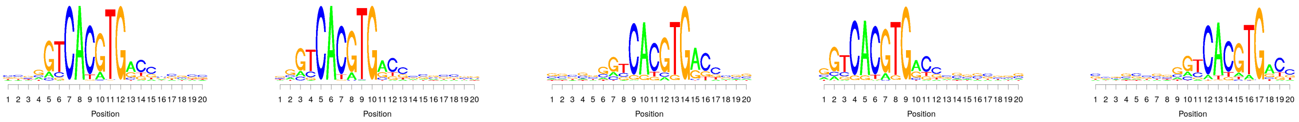

YY1 (3646 ChIP-Seq positives)

PWM	PMM1	PMM2	PMM3	PMM4

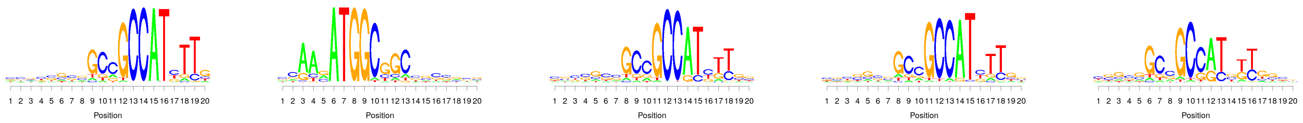

ZNF143 (6134 ChIP-Seq positives)

PWM	PMM1	PMM2	PMM3	PMM4

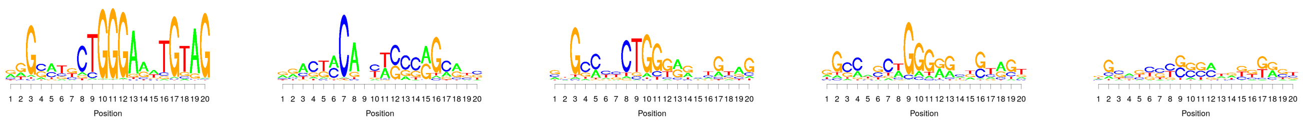

2 No meaningful motif identified

CHD1 (1380 ChIP-Seq positives)

PWM	PMM1	PMM2	PMM3	PMM4

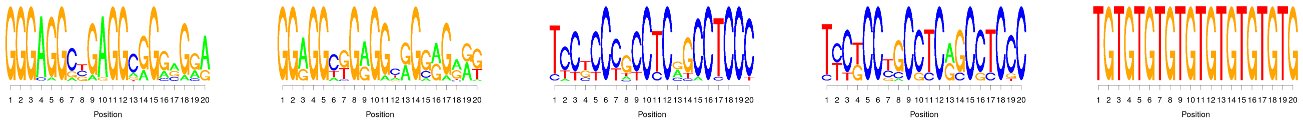

CTBP2 (1418 ChIP-Seq positives)

PWM	PMM1	PMM2	PMM3	PMM4

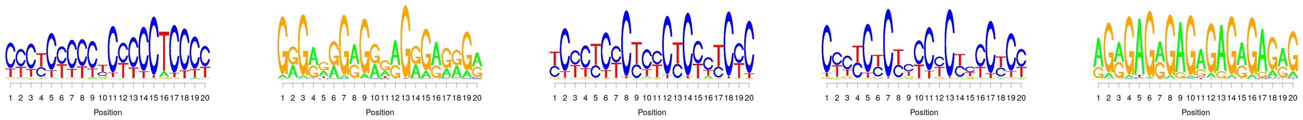

EP300 (1787 ChIP-Seq positives)

PWM	PMM1	PMM2	PMM3	PMM4

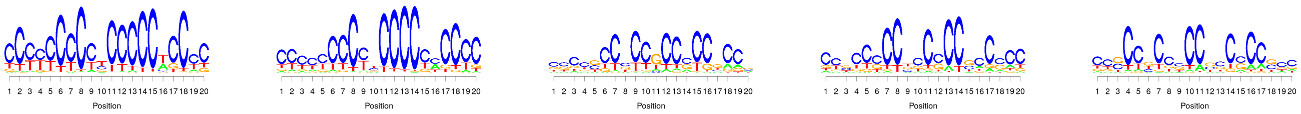

EZH2 (881 ChIP-Seq positives)

PWM	PMM1	PMM2	PMM3	PMM4

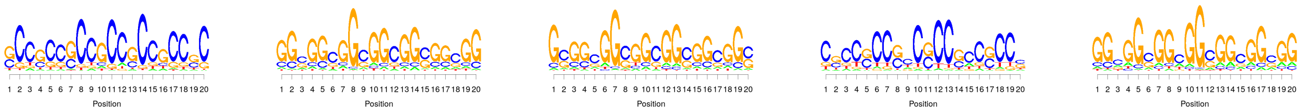

GTF2F1 (709 ChIP-Seq positives)

PWM	PMM1	PMM2	PMM3	PMM4

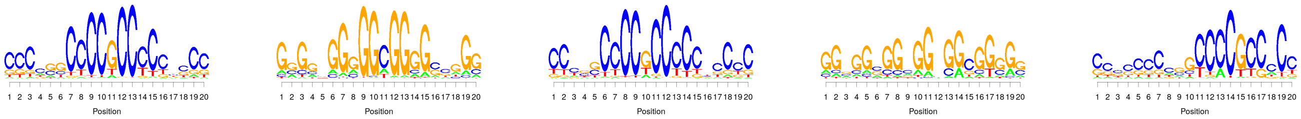

HDAC2 (1129 ChIP-Seq positives)

PWM	PMM1	PMM2	PMM3	PMM4

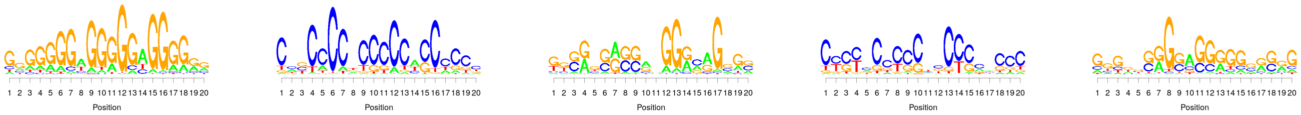

MYC (244 ChIP-Seq positives)

PWM	PMM1	PMM2	PMM3	PMM4

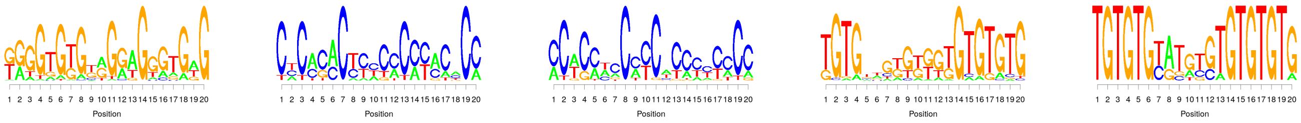

POLR2A (4077 ChIP-Seq positives)

PWM PMM1 PMM2 PMM3 PMM4

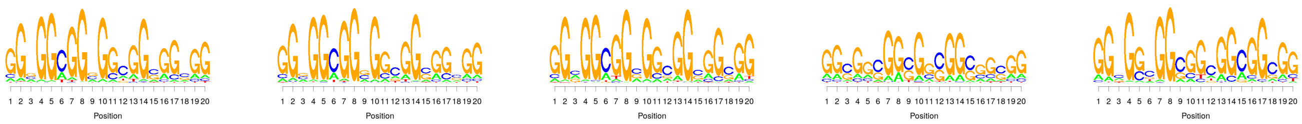

RBBP5 (3227 ChIP-Seq positives)

PWM PMM1 PMM2 PMM3 PMM4

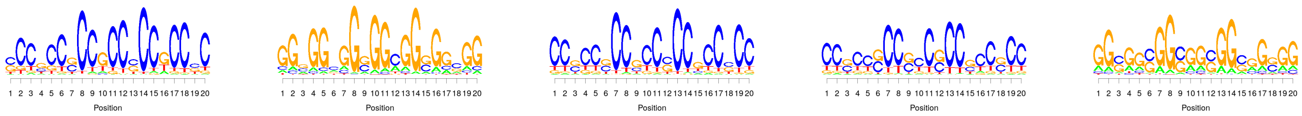

SIN3A (1796 ChIP-Seq positives)

PWM PMM1 PMM2 PMM3 PMM4

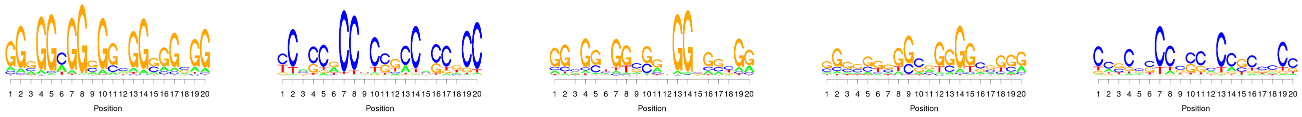

SUZ12 (958 ChIP-Seq positives)

PWM PMM1 PMM2 PMM3 PMM4

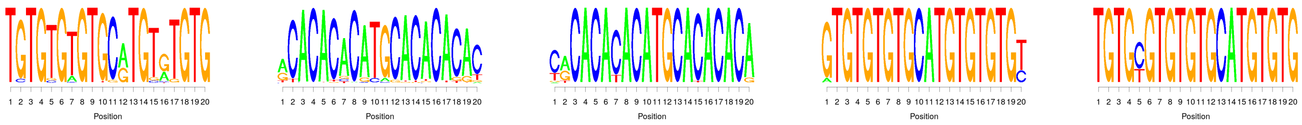

TAF1 (4008 ChIP-Seq positives)

PWM PMM1 PMM2 PMM3 PMM4

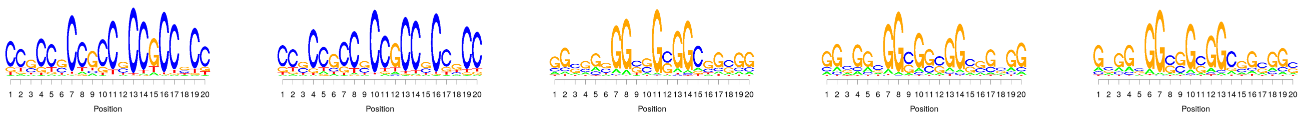

TAF7 (2072 ChIP-Seq positives)

PWM	PMM1	PMM2	PMM3	PMM4

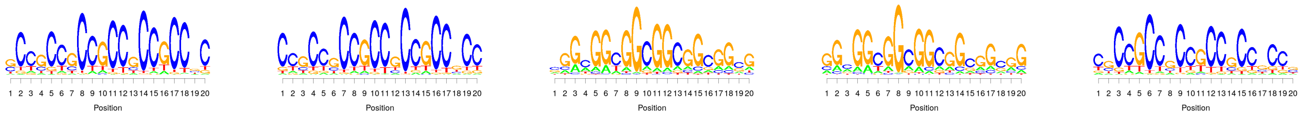

TBP (3429 ChIP-Seq positives)

PWM	PMM1	PMM2	PMM3	PMM4

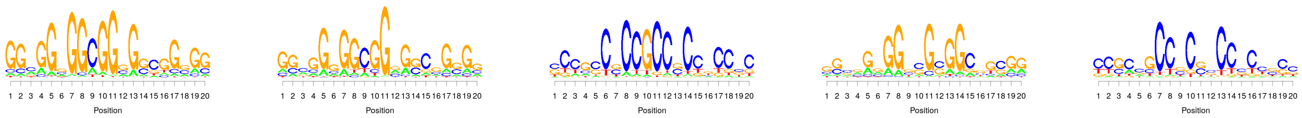

Supplement: Additional file 1 — Sequence logos of predicted binding sites. The file contains sequence logos of predicted binding sites for all 50 data sets and all five model orders. (PDF 4147.2 kb) [file 12859_2015_797_MOESM1_ESM.pdf]
