# Supplementary figures and images for: Inferring intra-motif dependencies of DNA binding sites from ChIP-seq data
Source: BMC Bioinformatics. 2015 Nov 9;16:375. doi: 10.1186/s12859-015-0797-4 (PMC4640111; doi:10.1186/s12859-015-0797-4)

Jenson-Shannon divergence

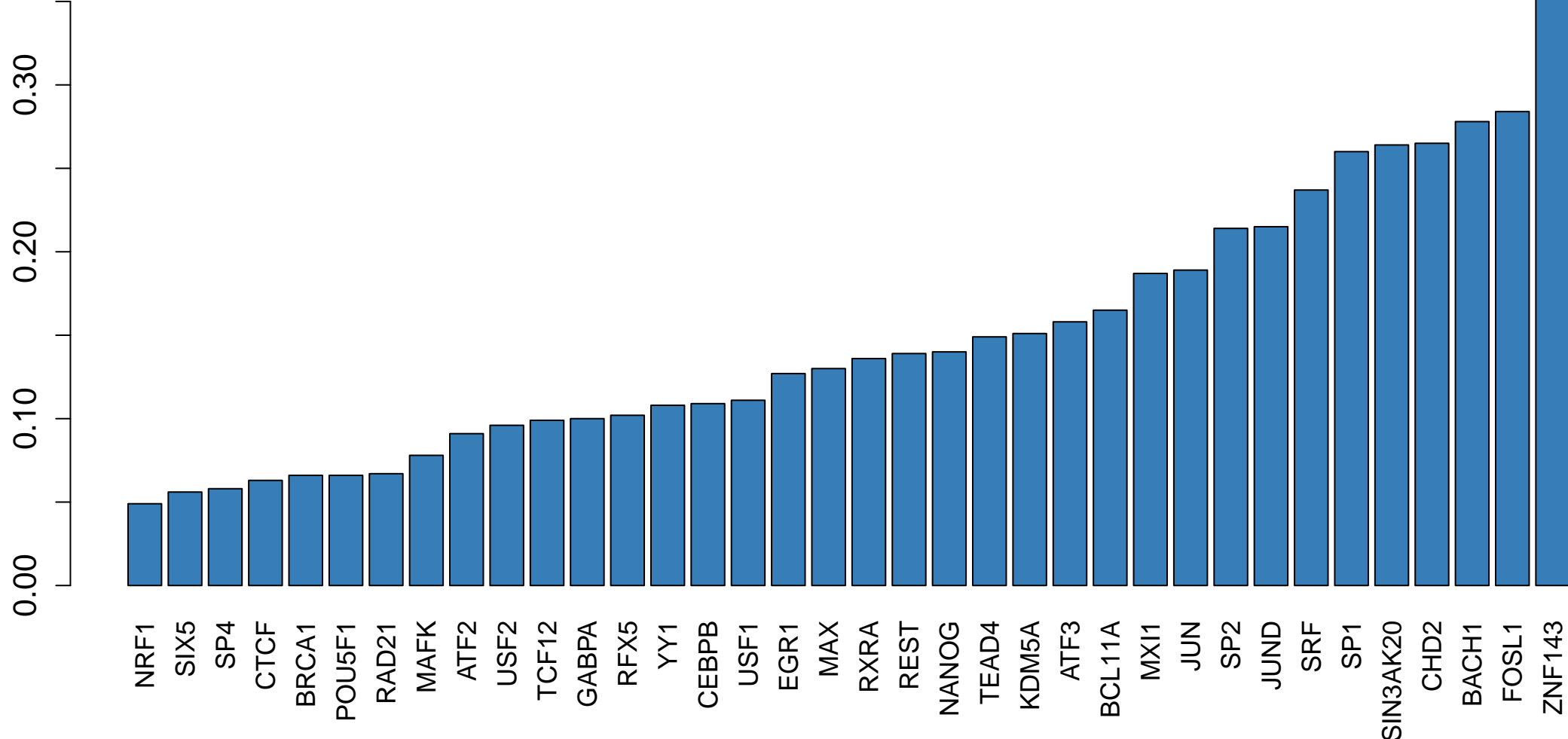

Supplement: Additional file 2 — Average JSD values. The plot shows the JSD values of the de-mixing experiment for all 36 data sets that contain sequence motifs other than repeats. (PDF 4.95 kb) [file 12859_2015_797_MOESM2_ESM.pdf]
