# Supplementary material for: Inferring intra-motif dependencies of DNA binding sites from ChIP-seq data
Source: BMC Bioinformatics. 2015 Nov 9;16:375. doi: 10.1186/s12859-015-0797-4 (PMC4640111; doi:10.1186/s12859-015-0797-4)
Supplement: Additional file 3 — p -values of model comparison. The tables show p-values of Wilcoxon signed ranked tests comparing the different models, one for each group of data sets. (PDF 26.3 kb) [file 12859_2015_797_MOESM3_ESM.pdf]

all data sets	PMM1	PMM2	PMM3	PMM4
PWM	**9.60E-10**	**2.91E-10**	**1.25E-09**	**9.60E-10**
PMM1		**2.33E-05**	**6.25E-04**	**1.36E-03**
PMM2			8.71E-01	8.82E-01
PMM3				8.82E-01

Category A	PMM1	PMM2	PMM3	PMM4
PWM	**1.13E-06**	**5.96E-07**	**2.53E-06**	**1.97E-06**
PMM1		**7.37E-03**	5.87E-02	9.57E-02
PMM2			6.26E-01	3.60E-02
PMM3				3.81E-01

Category B	PMM1	PMM2	PMM3	PMM4
PWM	**9.77E-04**	**9.77E-04**	**9.77E-4**	**9.77E-04**
PMM1		**1.95E-03**	**2.92E-03**	**2.92E-03**
PMM2			3.65E-01	4.13E-02
PMM3				4.65E-01
